# The Secretome Profiling of a Pediatric Airway Epithelium Infected with hRSV Identified Aberrant Apical/Basolateral Trafficking and Novel Immune Modulating (CXCL6, CXCL16, CSF3) and Antiviral (CEACAM1) Proteins*[Fn FN2]

**DOI:** 10.1074/mcp.RA119.001546

**Published:** 2020-02-19

**Authors:** Olivier Touzelet, Lindsay Broadbent, Stuart D. Armstrong, Waleed Aljabr, Elaine Cloutman-Green, Ultan F. Power, Julian A. Hiscox

**Affiliations:** ‡ Department of Infection Biology, Institute of Infection and Global Health, University of Liverpool, Liverpool L3 5RF, UK; § Wellcome-Wolfson Institute for Experimental Medicine, School of Medicine, Dentistry & Biomedical Sciences, Queens University Belfast, Belfast BT9 7BL, UK; ¶ NIHR Health Protection Research Unit in Emerging and Zoonotic Infections, University of Liverpool, L69 7BE, UK; ‖ Biomedical Research Administration, Research Centre, King Fahad Medical City, P.O. Box 59046 Riyadh 11252, Saudi Arabia; ** Microbiology, Virology and Infection Control, Level 4 Camelia Botnar Laboratory, Great Ormond Street Hospital, London WC1N 3JH, UK; ‡‡ Singapore Immunology Network, Agency for Science, Technology and Research (A*STAR), Singapore 138648, Singapore

**Keywords:** Cell secretion, cytokines, host-pathogen interaction, label-free quantification, lung function or biology, mass spectrometry, secretome, viruses, CEACAM1, CSF3, CXCL6, respiratory syncytial virus, well-differentiated pediatric bronchial epithelial cells (WD-PBECs)

## Abstract

The respiratory epithelium comprises polarized cells at the interface between the environment and airway tissues. Polarized apical and basolateral protein secretions are a feature of airway epithelium homeostasis. Human respiratory syncytial virus (hRSV) is a major human pathogen that primarily targets the respiratory epithelium. However, the consequences of hRSV infection on epithelium secretome polarity and content remain poorly understood. To investigate the hRSV-associated apical and basolateral secretomes, a proteomics approach was combined with an *ex vivo* pediatric human airway epithelial (HAE) model of hRSV infection (data are available via ProteomeXchange and can be accessed at https://www.ebi.ac.uk/pride/ with identifier PXD013661). Following infection, a skewing of apical/basolateral abundance ratios was identified for several individual proteins. Novel modulators of neutrophil and lymphocyte activation (CXCL6, CSF3, SECTM1 or CXCL16), and antiviral proteins (BST2 or CEACAM1) were detected in infected, but not in uninfected cultures. Importantly, CXCL6, CXCL16, CSF3 were also detected in nasopharyngeal aspirates (NPA) from hRSV-infected infants but not healthy controls. Furthermore, the antiviral activity of CEACAM1 against RSV was confirmed *in vitro* using BEAS-2B cells. hRSV infection disrupted the polarity of the pediatric respiratory epithelial secretome and was associated with immune modulating proteins (CXCL6, CXCL16, CSF3) never linked with this virus before. In addition, the antiviral activity of CEACAM1 against hRSV had also never been previously characterized. This study, therefore, provides novel insights into RSV pathogenesis and endogenous antiviral responses in pediatric airway epithelium.

## Abstract (precis)

This is the first comprehensive analysis of secretome in a hRSV-infected paediatric airway epithelium, which identified skewing of apical/basolateral abundance ratios for individual proteins, and validated three novel biomarkers (CXCL6, CXCL16 and CSF3) and a novel antiviral protein (CEACAM1).

## Graphical Abstract

**Figure:**
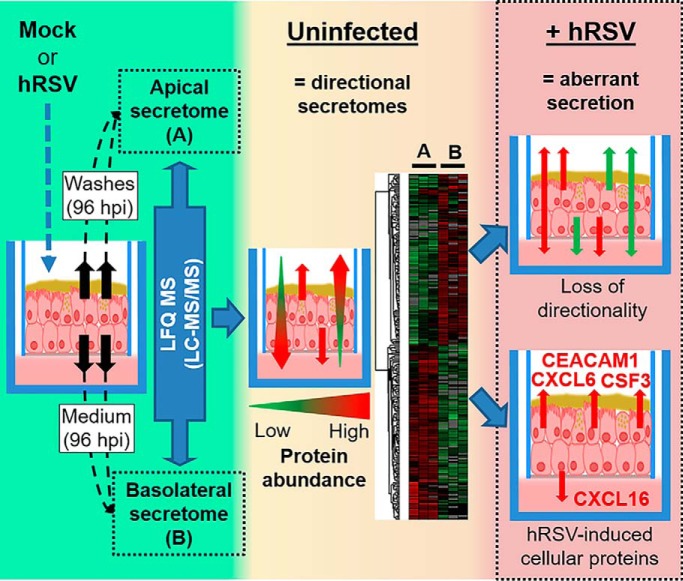

**Highlights**

- Proteome of airway secretions derived from mock- and hRSV-infected WD-PBEC cultures.
- A polarised secretome in uninfected WD-PBECs, skewed in hRSV-infected cultures.
- CXCL6, CXCL16, CECACAM1 and CSF3 induced only upon hRSV-infection.
- Detection of CXCL6, CXCL16 and CSF3 in NPAs from hRSV-positive children.

The epithelial lining fluid is essential in regulating the homeostasis of the airways. Apical secretions contain proteins associated with anti-oxidative, anti-protease, anti-microbial, and anti-inflammatory functions (1–5), and provide the first line of defense against inhaled particulates, including bacteria and viruses. Analysis of respiratory secretions by proteomics demonstrated alterations of the protein composition in chronic airway diseases, such as chronic obstructive pulmonary disease (COPD)^1^, asthma and cystic fibrosis (CF) (6–9). Moreover, proteome modifications were observed in bronchoalveolar lavage fluids (BALF) or nasopharyngeal aspirates (NPAs) from human immunodeficiency virus (HIV)-seropositive adults (10), and in hRSV-infected children (11), respectively. The respiratory epithelium basolateral side juxtaposes deeper lung tissues. Proteins secreted basolaterally impact on the lung sub-epithelial structures and cells, such as serous/mucous glands, fibroblasts, smooth muscle, blood vessels or immune cells. Thus, alterations of the apical and basolateral airway secretions are likely to reflect pathogenic states and play a key role in disease progression.

hRSV is a major cause of severe acute lower respiratory tract infection (ALRI) in young infants, the immunocompromised and the elderly. It has a high burden in children <5 years old, with ∼34 million new ALRI episodes each year and up to 200,000 deaths (12). hRSV primarily targets the respiratory epithelium and has been extensively characterized in a model of well-differentiated pediatric airway epithelial cell (HAE) cultures (13–20). In this system, the provision of an air-liquid interface to confluent human primary nasal or bronchial epithelial cells grown on solid permeable supports drives differentiation into pseudostratified columnar mucociliary epithelium that mimics the morphology, physiology and polarity of human respiratory epithelium *in vivo* (13–20). RSV infection of HAE cultures recapitulates many of the features associated with hRSV infection *in vivo* (reviewed in (21)). However, the impact of hRSV infection of the airway epithelium on the overall protein secretome is unknown. Therefore, the HAE model provides an ideal platform to address the proteome of airway secretions, thereby providing extensive information on the molecular changes associated with infection. Importantly, HAE cultures provide easily accessible apical and basolateral sides, thereby allowing analysis of airway secretions in both directions, in contrast to NPAs or BALF, which, for practical reasons can only inform changes associated with the apical-facing epithelial surface.

This study evaluated the impact of hRSV on airway epithelium secretions from pediatric HAE cultures, defined as well-differentiated pediatric primary bronchial epithelial cells (WD-PBECs), by characterizing the protein composition of the apical and basolateral secretomes upon infection relative to mock-infected control cultures, using quantitative proteomics (LC-MS/MS). Importantly, novel proteins associated with RSV infection were identified, including proteins with immune modulating or antiviral activities. Furthermore, our HAE findings reflected changes in NPAs from hRSV-infected infants.

## EXPERIMENTAL PROCEDURES

### 

#### 

##### Cells

HEp-2 cells were kindly provided by Ralph Tripp (University of Georgia) and grown at 37 °C with 5% CO_2_ in Dulbecco's modified Eagle's medium (DMEM) high glucose (4.5 g/L) supplemented with 5% (v/v) FBS and penicillin (100 IU/ml) and streptomycin (100 μg/ml) (Pen Strep, Life Technologies, UK). BEAS-2B cells (ATCC CRL-9609) were grown in DMEM low glucose (1 g/L) supplemented with 5% (v/v) FBS, penicillin (100 IU/ml) and streptomycin (100 μg/ml). Commercially available primary pediatric bronchial epithelial cells (donors aged 2–9 years) (Lonza, Switzerland) were grown on Transwells (Corning, NY; 6.5 mm diameter, 0.4 ?m pore size) as previously published (20) to generate well-differentiated pseudostratified cultures. The use of such commercial primary cells in this study did not require ethical approval. After initiating “air-liquid interface” culture, the basolateral medium (Promocell, UK) was replaced with fresh medium and any liquid or mucus on the apical surface was aspirated every 2 days. The apical side was washed prior to infection with 200 μl serum free (SF)-DMEM (no additives). Both the virus stocks and the cell lines were confirmed to be mycoplasma-free using Lookout mycoplasma PCR detection kit.

##### Virus

The hRSV BT2a clinical isolate was isolated from an infant hospitalized with bronchiolitis and passaged 3 times in HEp-2 cells (22). hRSV/eGFP was obtained from Prof. Ralph Tripp and Prof. Michael Teng (University of South Florida) and propagated in HEp-2 cells. Briefly, hRSV stocks were grown in HEp-2 cells (MOI 0.1) and harvested by cell scraping when extensive cytopathic effects (CPE) were obvious (80–90% of cells floating/rounded up and extensive syncytia). Following sonication (10 min, 4 °C, intensity 9) and clarification of the lysate at 3200 × *g* for 10 min at 4 °C the supernatant was aliquoted and snap-frozen and stored in liquid nitrogen.

##### RSV Titration - Tissue Culture Infectious Dose (TCID_50_)

RSV titers for virus stocks, in apical rinses and BEAS-2B supernatants were determined in HEp-2 cells. In brief, HEp-2 cells were seeded at 5 × 10^4^ cells/well on 24 well plates. The following day 200 μl of a 10-fold serial dilution of the virus stock, apical rinse or BEAS-2B supernatant were added to the HEp-2 cells in quadruplicate (from 10^−1^ to 10^−8^ dilutions). Following incubation for 2 h at 37 °C the inoculum was removed and 1 ml DMEM (High glucose) supplemented with 1% FBS (v/v) and penicillin (100 IU/ml) and streptomycin (100 μg/ml) was added. Cultures were incubated for 7 days at 37 °C and then examined for signs of CPE. Titers were calculated using the Kärber method and reported as log_10_ TCID_50_/ml (23).

##### RSV Infection of WD-PBECs

WD-PBEC cultures from each donor were infected with hRSV BT2a at a multiplicity of infection (MOI) of 0.1, using SF-DMEM (no additives)-treated cells as mock infection. To calculate the MOI cultures from one well from each donor were trypsinized and enumerated. Just before infection, the apical side was washed twice with 200 μl SF-DMEM (no additives) and fresh medium was added to the basolateral side. Cultures were infected on the apical side by incubation for 2 h at 37 °C 5% CO_2_ with 200 μl of virus stock diluted in SF-DMEM (no additives) beforehand, or with medium only (mock). Subsequently, the virus inoculum was removed, and the apical side was rinsed 4 times with SF-DMEM (no additives). The cultures were kept at 37 °C 5% CO_2_ and monitored daily by light microscopy for CPE.

##### Sample Collection from WD-PBECs

After infection, the basolateral medium (300 μl) and apical wash (200 μl) from hRSV BT2a- and mock-infected cultures were collected at 24, 48, 72, 96, and 120 h post-infection (hpi) for each donor, snap-frozen in liquid nitrogen and stored at −80 °C. The apical wash was generated by incubation with 200 μl of SF-DMEM (no additives) at RT for 5 min followed by gentle pipetting up and down three times, whereas fresh medium was added in the basolateral compartment. In order to remove cell debris and inactivate the virus before sample preparation for MS, the apical and basolateral samples were centrifuged at 3000 × *g* for 10 min at +4 °C and the supernatant was heat-treated for 10 min at 95 °C.

##### Sample Preparation for Label-free Mass Spectrometry

Apical (135 μl) and basolateral (180 μl) samples collected at 96 h post-infection (hpi) from control and hRSV-infected WD-PBEC cultures were prepared for analysis by NanoLC MS ESI MS/MS (LC-MS/MS). Soluble proteins were precipitated by addition of an equal volume of ice-cold 30% (w/v) trichloroacetic acid (TCA) in acetone with incubation at −20 °C for 2 h. Samples were centrifuged at 12,000 × *g* for 10 min (4 °C) to pellet proteins. Pellets were washed three times with ice-cold acetone and allowed to air dry. Protein pellets were then re-suspended in 50 mm ammonium bicarbonate, 0.1% (w/v) RapiGest SF (Waters, Elstree, UK). Samples were further processed and analyzed by NanoLC MS ESI MS/MS as previously described (24). Samples were heated at 80 °C for 10 min, reduced with 3 mm DTT at 60 °C for 10 min, cooled, then alkylated with 9 mm iodoacetamide (Sigma) for 30 min (room temperature) protected from light; all steps were performed with intermittent vortex-mixing. Proteomic-grade trypsin (Sigma) was added (0.2 μg) and incubated at 37 °C overnight. To remove RapiGest SF, the samples were precipitated using 1% (v/v) TFA at 37 °C for 2 h and centrifuged at 12,000 × *g* for 1 h (4 °C). The peptide supernatant was desalted using C_18_ reverse-phase stage tips (Thermo Scientific Pierce) according to the manufacturer's instructions. Samples were desalted and reduced to dryness and re-suspended in 3% (v/v) acetonitrile, 0.1% (v/v) TFA for analysis by MS. All reagents are supplied by Sigma-Aldrich unless stated otherwise.

##### NanoLC MS ESI MS/MS Analysis

Peptides were analyzed by on-line nanoflow LC using the Ultimate 3000 nano system (Dionex). Samples were loaded onto a trap column (Acclaim PepMap 100, 2 cm × 75 μm inner diameter, C18, 3 μm, 100 Å) at 9 μl/min with an aqueous solution containing 0.1% (v/v) TFA and 2% (v/v) acetonitrile. The trap column was set in-line an analytical column (Easy-Spray PepMap® RSLC 50 cm × 75 μm inner diameter, C18, 2 μm, 100 Å) fused to a silica nano-electrospray emitter (Dionex). The column was operated at a constant temperature of 35 °C and the LC system coupled to a Q-Exactive mass spectrometer (Thermo Fisher Scientific). Chromatography was performed with a buffer system consisting of 0.1% formic acid (buffer A) and 80% acetonitrile in 0.1% formic acid (buffer B). The peptides were separated by a linear gradient of 3.8–50% buffer B over 90 min at a flow rate of 300 nl/min. The Q-Exactive was operated in data-dependent mode with survey scans acquired at a resolution of 70,000 at *m*/*z* 200. Scan range was 300 to 2000 *m*/*z*. Up to the top 10 most abundant isotope patterns with charge states +2 to +5 from the survey scan were selected with an isolation window of 2.0 Th and fragmented by higher energy collisional dissociation with normalized collision energies of 30. The maximum ion injection times for the survey scan and the MS/MS scans were 250 and 50 ms, respectively, and the ion target value was set to 1E6 for survey scans and 1E5 for the MS/MS scans. MS/MS events were acquired at a resolution of 17,500. Repetitive sequencing of peptides was minimized through dynamic exclusion of the sequenced peptides for 20 s.

##### Protein Identification and Quantification

Label-free quantitation was performed using MaxQuant software (version1.5.3.3 (25)) with its internal search engine Andromeda (26). Precursor mass and fragment mass were searched with mass tolerance of 4.5 and 20 ppm respectively. All other settings were default. The search included variable modifications of oxidation (methionine) and protein N-terminal acetylation, and fixed modification of carbamidomethyl (cysteine). Enzyme specificity was set to trypsin (Trypsin/P), minimal peptide length was set to 7 amino acids and a maximum of two mis-cleavages were allowed. The false discovery rate (FDR) was set to 0.01 for peptide and protein identifications. The Andromeda search engine was configured for a database containing human proteins and hRSV (Uniprot release-2015_01, 20226 entries (27)). The software further includes a decoy database as well as common contaminants database to determine the false discovery rate and to exclude false positive hits because of contamination by proteins from different species. For label-free quantification (LFQ) analysis, “multiplicity” was set to one. Matching between runs was enabled. Only unmodified and unique peptides were utilized. The mass spectrometry proteomics data have been deposited to the ProteomeXchange Consortium via the PRIDE (28) partner repository with the data set identifier PXD013661. Parameter search and acceptance criteria, a summary of the raw mass spectrometric data, and lists of identified peptides and proteins are also included in supplemental Tables S1, S2, S3, and S4, respectively. Averaged LFQ intensity values were used to calculate protein ratios. Contaminants, keratins and host proteins only identified by unique peptides were excluded. Each protein was characterized by its percentage of sequence coverage, its molecular weight, the number of unique peptides used for identification and its abundance (label-free quantification [LFQ]) in the apical and basolateral samples. Proteins detected in at least 2 donor replicates, with at least two unique peptides per replicate, were retained for further analysis.

##### Prediction of the Apical and Basolateral Secretome

The secretome analysis followed an approach recently reported (29), which used several algorithmic methods to predict the presence of a signal peptide (SP) and/or a transmembrane region (TM) based on the amino acid sequence of a protein. In this study, SignalP4.1 (http://www.cbs.dtu.dk/services/SignalP/), an update from SignalP4.0 (30), Phobius (http://phobius.sbc.su.se/) (31), SPOCTOPUS (http://octopus.cbr.su.se/index.php) (32) and TMHMM2.0 (http://www.cbs.dtu.dk/services/TMHMM/) (33) were used to identify proteins predicted to carry SPs and/or TMs, and secretomeP2.0 (http://www.cbs.dtu.dk/services/SecretomeP/) (34) to distinguish proteins secreted in a non-classical fashion, all using default settings (35). SignalP4.1 and TMHMM2.0 only focused on the prediction of SP or TM, respectively, whereas Phobius and SPOCTOPUS combined the prediction of SP and TM (Phobius SP/TM and SPOCTOPUS SP/TM). All five algorithms are trained against specific data sets to discriminate between the type of protein feature or the secretory pathway involved. These programs were used to classify proteins following a majority-decision based method, whereby at least two out of three methods must predict that a protein carries a specific topological feature (supplemental Fig. S3). Therefore, all identified proteins in the apical wash and basolateral medium of mock- and hRSV BT2a-infected WD-PBEC cultures were organized accordingly as secreted via a signal peptide, secreted in a non-classical pathway, membrane or intracellular proteins. When required, exosome analysis was performed by searching the Exocarta database (http://www.exocarta.org/) (36).

##### Subjects and Clinical Specimen Collection

Nasopharyngeal aspirates (NPAs) were collected from six hRSVA- and six hRSVB-positive children (<1–9 years-old, median age = 2.5, IQR = 2.5) admitted to Great Ormond Street Hospital for children in London. hRSV diagnosis was confirmed by the Film Array multiplex polymerase chain reaction (PCR) system respiratory panel (Biomerieux diagnostics, Basingstoke, UK). This panel detects the 20 most common respiratory pathogens; adenovirus, coronavirus HKU1, coronavirus NL63, coronavirus OC43, human metapneumovirus, human rhinovirus/Enterovirus, influenza A, influenza A H1, influenza A H1–2009, influenza A/H3, influenza B, parainfluenza virus 1, parainfluenza virus 2, parainfluenza virus 3, parainfluenza virus 4, HRSV, *Bordetella pertussis*, *Chlamydophila pneumoniae*, and *Mycoplasma pneumoniae*. For this study they represented discarded samples and there was no identifying information (supplemental Table S5), and as such did not require ethical approval. Twelve NPA samples from hRSV-negative children of similar age (<1–9 years-old, median age = 2, IQR = 2.25), obtained during the same hRSV season, were used as controls. The NPA sampling was carried out as per the hospital's guidelines. Briefly, a catheter was inserted into the nose, directed posteriorly and toward the opening of the external ear, in order to reach the posterior pharynx. Suction was applied and the catheter was slowly withdrawn using a rotating movement, remaining less than 10 s in the nasopharynx. The catheter was then rinsed with a small volume of sterile 0.9% saline solution to ensure adequate specimen volume. Samples were stored at −80 °C until use.

##### Immunofluorescence of WD-PBEC Cultures

Immunofluorescence staining (IF) was carried out as previously described (20). Briefly, at 120 hpi the cultures were fixed with PBS +4% paraformaldehyde (v/v; Sigma-Aldrich) for 20 min. Fixed WD-PBEC cultures were stored in 70% ethanol (v/v in dH_2_O) at 4 °C until used. The cultures were rinsed 1× with PBS (pH 7.4) and permeabilized with PBS 0.2% Triton X-100 (v/v; ThermoFisher Scientific, UK) for 1 h at RT. After blocking for 30 min with 0.4% BSA (v/v in PBS, pH7.4) (Sigma Aldrich), cultures were stained with anti-β-tubulin Cy3-conjugated monoclonal antibody (ciliated cell marker) (Abcam, ab11309) diluted at 1:200, and anti-RSV F 488-conjugated antibody (Millipore, Nottingham, UK, MAB8262X) diluted at 1:500, incubated for 1 h at 37 °C. Goblet cells were stained with a rabbit anti-Muc5ac (Abcam, UK, ab78660) diluted at 1:100 and incubated overnight at 4 °C, followed by a goat anti-rabbit IgG conjugated to Alexa fluor 647 (Thermofisher, A21245), diluted at 1:500 and incubated 1h at 37 °C. All antibodies were diluted in 0.4% BSA. Following treatment with DAPI-mounting medium (Vectashield, Vector Labs, UK), fluorescence was detected in the cultures by UV microscopy (Nikon Eclipse 90i). Total RSV-infected and ciliated cells were enumerated in 5 fields of view per culture at ×60 magnification.

##### Western Blotting and ELISA

Apical and basolateral samples from control and infected WD-PBEC cultures, and NPAs were clarified at 3000 × *g* for 10 min at 4 °C. Protein concentration in NPA samples was estimated by Pierce^TM^ BCA assay (Thermofisher scientific). Twelve μl/lane of each sample from hRSV BT2a-infected cultures, a pooled sample from mock-infected cells (4 μl each), and 10 μg/lane of NPAs were then resolved by 10 or 12% SDS-PAGE and transferred to PVDF membranes (Millipore) using a Bio-Rad semi-dry transfer apparatus. An aliquot of sucrose-gradient purified hRSV A2 strain or a cell lysate from HEp-2 cells infected with hRSV A2 (MOI = 1) for 24 h were used as positive controls. Immobilized host and virus proteins were detected with the following primary antibodies: rabbit anti-CSF3 (ab181053, Abcam), goat anti-hRSV proteins (ab20745, Abcam), mouse anti-hRSV matrix protein (M024, 1:500) and nucleoprotein (mab0015, 1:500), both kindly provided by Prof. Paul Yeo, Durham University, mouse anti-hRSV M2-1 (ab94805, Abcam) and mouse anti-hRSV Fusion protein (ab94968, Abcam). Unless stated, all primary antibodies were diluted 1:1000 in tris-buffered saline supplemented with 2% milk. Two HRP-conjugated secondary antibodies (goat anti-rabbit IgG [A6154] and anti-mouse IgG [A4416]) were obtained from Sigma and one from Abcam (rabbit anti-goat IgG [ab6741]). All were detected by enhanced chemiluminescence (ECL) with the Clarity™ Western ECL Blotting Substrate from Bio-Rad (Hemel Hempstead, UK). In all cases, the apparent molecular weights were estimated using Color Prestained Protein Standard (11–245 kDa) from New England Biolabs (Ipswitch, UK). CXCL6 and CXCL16 were quantified with Human Quantikine ELISA Kits (R&D systems, Abingdon, UK), as per suppliers' recommendations.

##### Functional Assay

A polyclonal rabbit anti-CEACAM1 antibody (Agilent, Stockport, UK, IS526530), known to block its activity (37, 38), and a polyclonal rabbit isotype control antibody (R&D, AB-105-C) were dialyzed in PBS to remove any traces of sodium azide, as per manufacturer's instructions (Slide-a-lyser cassettes, MWCO = 10K, #66380, ThermoFisher Scientific). Dilutions of antibodies were prepared in PBS with dilution factors of 1:2, 1:3, 1:5, 1:10, and 1:100 from the dialyzed stocks. BEAS-2B cells were seeded in 96-well plates at 10^4^ cells/well the day before infection and infected in triplicate (50 μl/well) with combinations of hRSV/eGFP (MOI = 3) and dilutions of the antibodies/PBS. After two h of virus adsorption, cells were washed once with PBS (100 μl/well) and incubated for 72 h in 100 μl/well of DMEM (supplemented with 1% FBS and pencillin/streptomycin) containing the appropriate dilution of antibodies/PBS. The infection was followed by fluorescence imaging every 24 hpi (3 pictures taken per well) and the fluorescence was analyzed with ImageJ software (fluorescence intensity and percentage of eGFP coverage per field view). Supernatant (25 μl/well) was also collected every 24 hpi and virus titration carried out by TCID_50_. Twenty five μl/well were replaced with fresh medium containing the appropriate antibody/PBS treatment.

##### Experimental Design and Statistical Rationale

For LC-MS/MS analysis, three independent biological replicate experiments were performed using WD-PBECs derived from three individual donors. For each donor, apical washes and basolateral medium were collected from WD-PBECS cultures, which were either mock-treated (mock) or infected with hRSV BT2a. Therefore, a total of 12 samples (collected at 96 hpi) were analyzed by LC-MS/MS.

All bioinformatics analyses of protein abundance were performed using PERSEUS 1.6.1.3 software. For comparative analysis protein abundance values were log_2_-transformed. The significantly enriched apical and basolateral proteins were highlighted with volcano plots (two-sample *t* test with a permutation-based FDR method) and further selected using an adjusted *p* value <0.05 and a fold change >2 as filtering criteria. Log2-transformed individual values or triplicate means were z-score-normalized prior to hierarchical clustering. Gene ontology biological process (GOBP) enrichment analysis was achieved with a Fisher exact test (Benjamini-Hochberg FDR 0.2%) and the cellular component enrichment analysis (GOCC) was achieved with a Fisher exact test (*p* value < 0.01). Exosome analysis was performed by searching the Exocarta database (http://www.exocarta.org/). When required, classification of proteins in BPs was achieved with Funrich v3.0 software.

Each apical and basolateral sample tested by LC-MS/MS (derived from each donor and either mock-treated or infected) was also validated by Western blotting, whereas the corresponding cultures (fixed at 120 hpi) were analyzed by immunofluorescence. For ELISA quantification in samples from WD-PBECs cultures, statistical analysis was performed with a two-tailed paired Student's *t* test, and for the NPAs from healthy and hRSV-positive children a Mann-Whitney test was used. In the case of the CEACAM1 functional assay, statistical analysis was carried out with a 2-way ANOVA test and the results presented are representative of three independent experiments. For all statistical tests utilized *p* values <0.05 were considered significant.

## RESULTS

### 

#### 

##### hRSV Growth Kinetics, Cell Tropism, and Viral Protein Detection

**Fig. 1.**
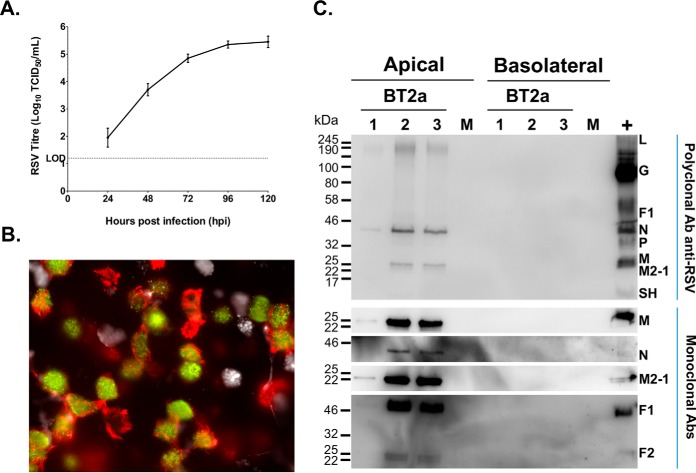
**hRSV targets ciliated cells but not goblet cells, and leads to a productive infection.** *A*, WD-PBEC cultures (*n* = 3 donors, in duplicate) were infected with hRSV (MOI = 0.1) or mock infected. Apical rinses were taken every 24 h and titrated using HEp-2 cells by TCID_50_. *B*, At 120 h post-infection (hpi) cultures were fixed with 4% paraformaldehyde, ciliated, goblet and infected cells were stained with anti-β-tubulin (red), anti-Muc5ac (white) and FITC-conjugated anti-RSV (green) antibodies, respectively. Cultures were examined by fluorescent microscopy at x63 magnification. *C*, Apical washes and basolateral medium collected at 96 hpi from infected cultures, and pooled samples from mock-infected cultures were analyzed individually by Western blotting (12 μl/lane). Ten μl/lane of a sucrose-gradient purified hRSV A2 stock was used as control. Immunoblot analyses was carried out using a polyclonal anti-hRSV antibody (upper panel) and monoclonal antibodies specific for the matrix protein (M), nucleoprotein (N), fusion (F) and M2-1 (lower panel), followed by appropriate HRP-conjugated secondary antibodies for enhanced chemiluminescence detection.

WD-PBEC cultures (*n* = 3 donors) were infected with hRSV BT2a (MOI = 0.1) or mock infected. hRSV growth kinetics, cell tropism and cytopathogenesis (CPE) were determined. Virus release was detected in apical rinses at 24 hpi and peaked at 96 hpi (Fig. 1*A*). hRSV primarily infected ciliated cells but not goblet cells (Fig. 1*B* and supplemental Fig. S1). There was no obvious CPE observed in cultures from any donor (data not shown). To further extend these findings, the presence of viral proteins was investigated by LC-MS/MS analysis of apical and basolateral samples collected at 96 hpi from mock- and hRSV-infected cultures. Five viral proteins were identified by at least two unique peptides, in infected cultures only from all donors. These included the hRSV matrix (M), fusion (F0), phosphoprotein (P), nucleoprotein (N), M2-1 proteins, in order of abundance (Table I). The RNA-dependent RNA polymerase (L) protein was detected in samples from 2 donors. In general, detection was restricted to the apical side, and confirmed by Western blotting analyses for viral proteins (Fig. 1*C*). Unexpectedly, hRSV M protein was detected by LC-MS/MS in the basolateral medium from one donor (Table I), but was not confirmed by Western blotting. Overall, these findings were consistent with the viral growth kinetics and the strict apical release of virions previously reported for hRSV in HAE models of adult or pediatric origin, and for both nasal and bronchial cells (13–19).

**Table I:** hRSV proteins detected by LC-MS/MS at 96 hpi in the apical washes or basolateral medium of hRSV-infected WD-PBECs

Protein name	Apical			Basolateral	
	% protein abundance^a^/replicates^b^	Unique peptides^c^	% cov.^d^	LFQ^e^/replicates^b^	Unique peptides^c^
Matrix	63.5/3	25	81.2	24.36/1	1
Fusion (F0)	11.6/3	25	53.3	N.D.^f^	N.D.
Phosphoprotein	9.3/3	12	85.9	N.D.	N.D.
Nucleoprotein	9.4/3	22	64.7	N.D.	N.D.
Matrix M2-1	5.9/3	5	60.3	N.D.	N.D.
RNA-dependent RNA polymerase L	0.38/2	4	4.4	N.D.	N.D.

^a^Percentage of the total abundance of all the viral proteins detected, averaged between replicates

^b^Number of replicates the protein was identified in

^c^Number of unique peptides used for protein identification

^d^The percentage of sequence coverage for the identified protein

^e^Abundance intensity expressed as Log_2_

^f^ N.D. = not detected

##### Characterization of the Secretomes from Control and hRSV-infected Cultures

**Fig. 2.**
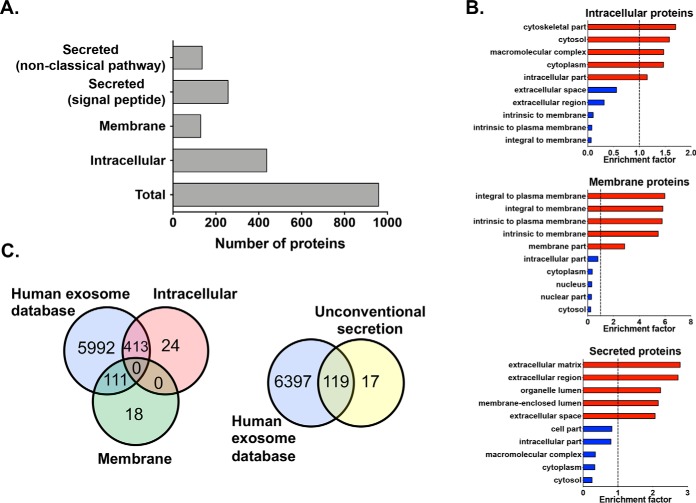
**Classification of proteins by predicted topology, GOCC enrichment and exosome analyze.** *A*, Number of proteins for each category (*x* axis). Using predictive software, all the proteins identified in mock- and hRSV-infected WD-PBEC cultures of each patient (*n* = 3) were combined and categorized into proteins secreted via a signal peptide or in a non-classical pathway, and cellular proteins with either intracellular or membrane localization. *B*, GOCC enrichment of the proteins predicted to be intracellular, membrane or secreted (classical and non-classical fashion), which identified the five most significantly enriched (>1, red bars) and depleted (<1, blue bars) cellular compartments in each protein group. *x* axis, gene ontology enrichment factor. Only GO classifications with a FDR < 1% are shown. *C*, Venn-diagrams showing the overlap between several categorises of secretome proteins and a human exosome database (Exocarta, http://www.exocarta.org/).

The apical and basolateral secretomes were determined by LC-MS/MS analysis of samples collected from mock- and hRSV-infected WD-PBEC cultures (96 hpi). A total of 959 host proteins were identified by at least 2 unique peptides and in cultures from ≥2 donors (supplemental Table S6). Unexpectedly, based on their predicted topology, intracellular (*n* = 437) and membrane (*n* = 129) proteins represented more than half the protein pool (Figs. 2*A* and supplemental Fig. S3), as opposed to mainly secreted proteins (*n* = 257, signal peptide; *n* = 136 non-classical pathway), and this was also observed in the apical and basolateral sides separately (supplemental Fig. S4). A GOCC enrichment analysis (Fisher exact test, Benjamini-hochberg FDR 0.1%) further validated this finding. In control and infected cultures, proteins predicted to be intracellular, membrane or secreted were mostly enriched in proteins assigned to cell parts/cytoplasm, the plasma membrane or the extracellular space/vesicle lumen, respectively, and the converse was true with regards to the depleted proteins (Fig. 2*B*). Although intriguing, the detection of many intracellular, membrane and unconventionally secreted proteins in apical and basolateral secretions was in line with other reports (1, 3, 5, 39). Their presence could be a consequence of cell sloughing, particularly in the apical side during hRSV infection (14–17), or of exosomal secretion, which is a feature of differentiated HAE (2) and hRSV-infected undifferentiated epithelial cells from the bronchial and small airway (40). Indeed, an overlap with a human exosome database (*n* = 6516, http://www.exocarta.org/) revealed that 413 intracellular (out of 437), 111 membrane (out of 129) and 119 (out of 136) unconventionally secreted proteins had been identified in previous exosomal data sets (Fig. 2*C*). This is consistent with the presence of these proteins in the basolateral secretome, considering the physical separation between cells and medium.

##### Secretome Directionality and Homeostatic Processes

**Fig. 3.**
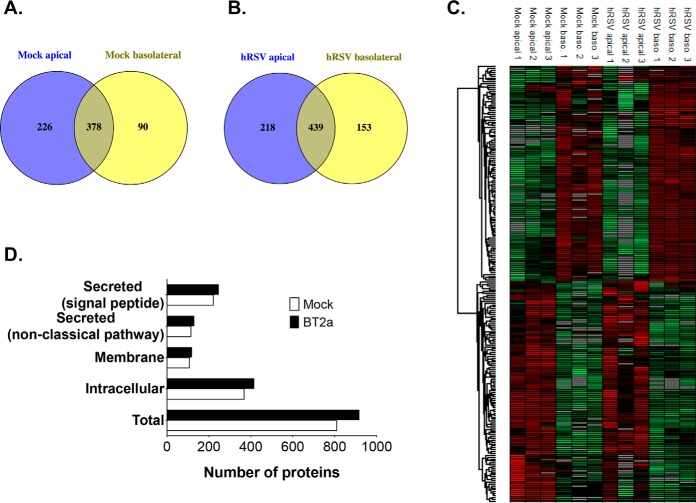
**The secretome of WD-PBECs cultures is directional.** *A* and *B*, Venn diagrams showing the distribution of apical and basolateral unique proteins in control (*A*) and infected (*B*) WD-PBECs cultures. *C*, Hierarchical clustering of proteins whose abundance was significantly changed between the apical and basolateral sides of mock and infected cultures (*p* value <0.05, fold change >2). Only the 229 proteins identified by volcano plot analysis are shown (supplemental Fig. S5). Log-2 transformed abundance values were Z-score-normalized for each replicate, which is a measure of the deviation from the sample mean. The hierarchical clustering was performed with the rows indicating individual proteins and the columns individual replicate (green, low abundance; red, high abundance; gray, undetected). See supplemental Fig. S6 for more detailed heatmaps of labeled proteins. *D*, Using predictive software (see supplemental Fig. S3), proteins from mock- and hRSV-infected WD-PBEC cultures (black and white bars, respectively) of each patient (*n* = 3) were categorized based on their expected topologies (*y* axis), and the total number in each category is shown (*x* axis).

To assess whether the polarity of healthy WD-PBEC cultures was reflected by their protein composition, apical and basolateral secretomes were compared with one another. In control cultures, 226 apical and 90 basolateral proteins were found to be unique (Fig. 3*A*), whereas a volcano plot analysis revealed that 157 proteins were significantly enriched in one side or the other (*p* < 0.05, supplemental Fig. S5*A*). A hierarchical clustering of the abundance patterns clearly validated this dichotomy (Fig. 3*C* and supplemental Fig. S6*A*), confirming a polarized secretome. Interestingly, basic homeostatic processes were distinct between the apical and basolateral secretomes of uninfected cultures, as revealed by a GOBP enrichment analysis performed on unique and enriched proteins (Fisher exact test, *p* value < 0.01). The apical secretome was significantly associated with proteins involved in lipid metabolism, o-glycosylation, signaling, transport, regulation of cell activation, calcium/ion homeostasis, response to stimulus, regulation of enzymes activity, sugar metabolism, cellular processes and other BPs (supplemental Fig. S7*A* and S7*B*). In contrast, the basolateral proteins were significantly associated with the regulation of metabolic and cellular processes, the regulation of cell death, cell contraction/movement and various metabolic and other pathways (supplemental Fig. S7*C* and S7*D*). Hence, under homeostatic conditions the airway secretome from WD-PBECs cultures was polarized in protein composition and biological functions, thus corroborating previous observations in HAE cultures (5).

##### Impact of hRSV on WD-PBEC Culture Secretomes

To determine whether hRSV impacted normal airway epithelial functions, the protein composition and directionality of the secretomes were compared between mock and infected cultures. More proteins were detected upon infection (*n* = 912) compared with control cultures (*n* = 810) (Fig. 3*D*), and this increase was more pronounced in the basolateral side (supplemental Figs. S4). Interestingly, the number of uniquely apical or basolateral proteins was also altered in hRSV-infected cultures (Fig. 3*A* and 3*B*), as was the number of significantly enriched proteins on either side (supplemental Fig. S5*B*).

**Fig. 4.**
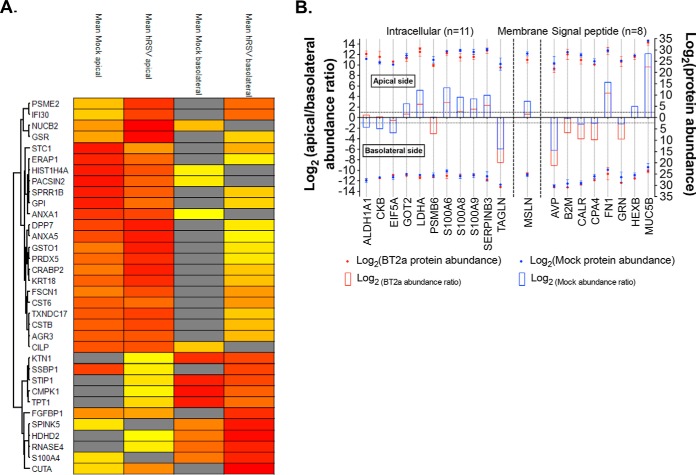
**Host proteins with secretion patterns most affected by hRSV infection.** *A*, Hierarchical clustering of proteins whose polarity of secretion was changed upon infection (*n* = 34). Log-2 transformed mean abundance values between replicates were Z-score-normalized. The hierarchical clustering was performed with the rows indicating individual proteins and the columns averaged replicate (yellow, low abundance; red, high abundance; gray, undetected). *B*, Graphical representation of the host proteins whose abundance ratios were the most disrupted upon infection (*n* = 20, delta fold change >2, see supplemental Table S6). For each protein (*x* axis), the bars represent the apical/basolateral ratios (left *y* axis) and the dots indicate the corresponding abundance (right *y* axis), in control (blue) and virus-infected (red) WD-PBEC cultures. Both protein abundance (LFQ intensity) and abundance ratios were plotted on a Log_2_ scale. Proteins were separated based on their predicted cellular location and the data were displayed for the apical (top) and basolateral (bottom) side.

In view of these observations and as directional protein secretion is characteristic of a healthy epithelium, apical/basolateral protein abundance ratios were calculated to determine disruptions in secretion patterns associated with RSV-infected airway epithelium (supplemental Fig. S8). Interestingly, 34 proteins were identified with altered secretion patterns, whereby their polarity of secretion was changed in infected cultures (Fig. 4*A*). Altered abundance ratios were also observed for several of the proteins significantly enriched under normal homeostatic conditions (Fig. 4*B* and supplemental Table S6). Moreover, many of those affected proteins were associated with biological processes (BPs) enriched under healthy conditions (supplemental Table S7). For instance in the apical side, affected BP groups included calcium/ion homeostasis, lipid or sugar metabolism, signaling, and responses to chemical or mechanical stimuli. Similarly, altered basolateral proteins were implicated mainly in various cellular/metabolic processes and their regulation, and the regulation of cell death. These data suggested that hRSV perturbed several homeostatic BPs by affecting the protein equilibrium between apical and basolateral sides and, by extension, disrupted normal airway epithelium functions.

**Fig. 5.**
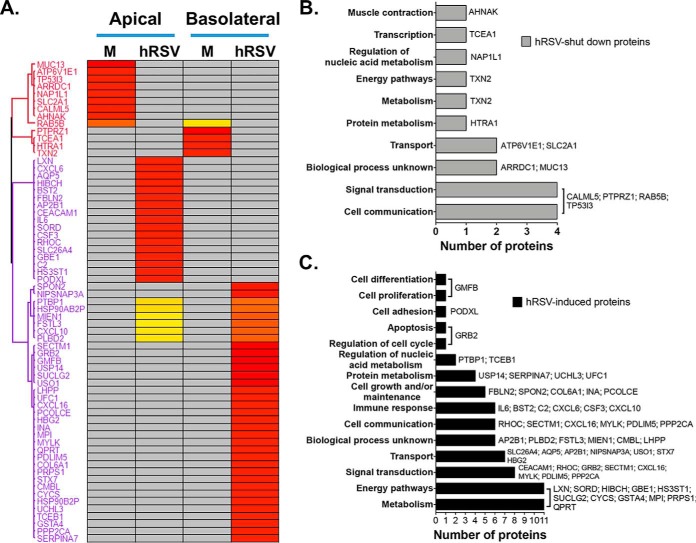
**Host proteins whose secretion was specifically induced (*n* = 53) or shut down (*n* = 13) upon infection.** *A*, The hierarchical clustering was performed with log-2 transformed abundance values, which were averaged for each group then Z-score-normalized. The rows indicate individual proteins and the columns individual replicate (yellow, low abundance; red, high abundance; gray, undetected). *B*, *C*, Classification of shut down (*B*) and induced (*C*) proteins based on their associated biological processes (BPs, *y* axis) and the number of proteins in each BP (*x* axis). Grouping was achieved with Funrich v3.0 and proteins are labeled for each BP.

**Fig. 6.**
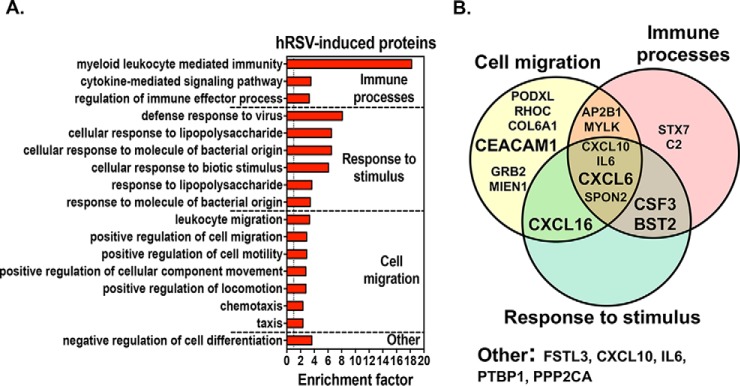
**Enriched BP associated with hRSV-induced proteins.** *A*, The most significantly enriched BP (>1, *y* axis) associated with hRSV-induced proteins were identified by GOBP enrichment, and further divided in functional groups. *x* axis, gene ontology enrichment factor. Only GO classifications with a *p* value <0.01 are shown. *B*, Venn-diagrams showing the overlap between the hRSV-induced proteins specifically associated with the enriched BPs (*n* = 17) and the four functional groups.

In addition to these exciting observations, a further 65 host proteins were specifically induced (*n* = 52) or no longer detectable (*n* = 13) at 96 hpi (Fig. 5*A* and supplemental Table S6). Functional classification showed that most of the 13 undetectable proteins were involved in physiological processes, such as cell communication, signal transduction and transport (Fig. 5*B*). Of note, groups of BPs involving calcium/ion transport and o-glycosylation were affected via the loss of SLC2A1/ATP6V1E1 and MUC13, respectively (supplemental Table S7). hRSV-induced proteins were mainly implicated in energy pathways, metabolism, signal transduction, cell communication and immune responses (Fig. 5*C*). Of these, 17 proteins were associated with significantly enriched BPs indicative of viral infection: immune processes, response to stimulus, cell migration and the negative regulation of cell differentiation (Fig. 6 and supplemental Table S9). They included several neutrophil (CXCL6, CXCL10, and IL6) and lung T cell (CXCL16) chemoattractants (41), a neutrophil growth factor (CSF3) and 2 proteins with antiviral properties against Influenza or hMPV (BST2 and CEACAM1) (37, 38, 42). In addition, SECTM1, which is implicated in signal transduction and cell communication (Fig. 5*C*), was previously identified as a neutrophil activator (43). As a major pathological component of hRSV-induced disease is the large infiltration of neutrophils in the lungs (44), these data are extremely relevant in the context of hRSV pathophysiology. In an exciting development, whereas CXCL10 and IL6 were previously associated with responses to hRSV infection in WD-PBECs (15) and children (45, 46), an association between hRSV infection and CXCL6, CSF3, CXCL16, SECTM1 and BST2 is a novel observation. Importantly, these novel associations were confirmed to be strictly infection-specific, as none of these proteins were present in the proteome of airway secretions (supplemental Table S10) from healthy patients (47) or uninfected HAE cultures (1–3, 5, 39).

##### Quantification of CXCL6 and CXCL16 by ELISA and Validation of CEACAM1 as a Novel hRSV Antiviral Factor

**Fig. 7.**
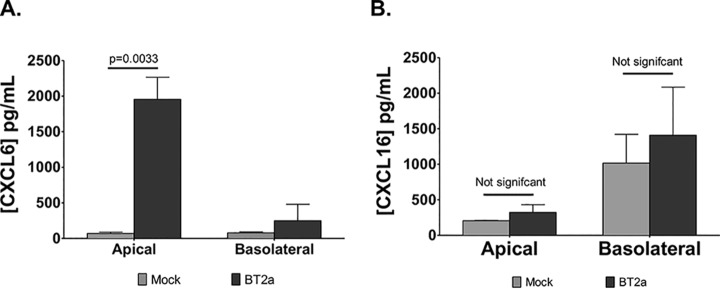
**CXCL6 and CXCL16 secretions increased following RSV infection of WD-PBECs.** Apical CXCL6 (*A*) and basolateral CXCL16 (*B*) concentrations were determined by ELISA at 96 hpi from mock- and hRSV-infected WD-PBECs (*n* = 3; mean ± S.D.). A *p* value <0.05 indicated statistical significance (Student's *t* test).

Neutrophil and T cell infiltrations are hallmarks of hRSV pathogenesis in infants. As CXCL6 and CXCL16 are chemotactic for neutrophils and T cells, respectively, validating our secretome data was imperative to further support a potential role for these proteins in hRSV pathogenesis. Therefore, changes in the abundance of CXCL6 and CXCL16 were confirmed by ELISA. CXCL6 concentrations were significantly increased in the apical side of infected WD-PBECs compared with mock-infected cultures (Fig. 7*A*). CXCL16 was more abundant in the basolateral side under homeostatic conditions and this difference was amplified following hRSV infection (Fig. 7*B*).

**Fig. 8.**
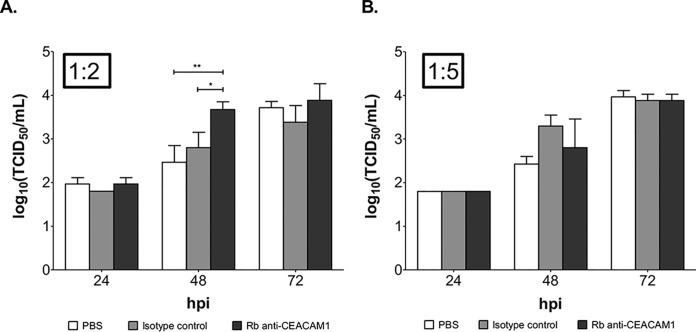
**Blocking CEACAM1 activity increases hRSV release in BEAS-2B.** hRSV titers in the supernatant of infected BEAS-2B cells (MOI = 3), which were treated with different dilutions of anti-CEACAM1 blocking Ab, an isotype Ab control or PBS, during the course of the infection (*x* axis). Dilution 1:2 (*A*) and 1:5 (*B*) are shown and titers were expressed as Log_10_ (TCID50/ml) (*y* axis). Statistical analysis was carried out with a 2-way ANOVA test and *p* values <0.05 were considered significant (*, *p* < 0.05; **, *p* < 0.01). The results presented are representative of three independent experiments.

CEACAM1 has both transmembrane and secreted isoforms, is up-regulated upon hRSV infection in A549 and undifferentiated primary bronchial epithelial cells, and displays an antiviral activity against influenza virus and hMPV (37, 38, 48, 49), a close relative of hRSV. To determine whether CEACAM1 has antiviral activity against hRSV, BEAS-2B cells were infected with hRSV/eGFP (MOI = 3) in the presence or absence of serial dilutions of an antibody that blocks CEACAM1 function (37, 38). BEAS-2B is a more relevant epithelial cell line (compared with A549) to study RSV and has been shown to express CEACAM1 (49, 50). Anti-CEACAM1 treatment led to a significant increase in fluorescence intensity supplemental Fig. S9*A*, S9*B*, and S9*C*) and percentage of fluorescence coverage of the monolayer (supplemental Fig. S9*D* and S9*E*) which resulted in an accelerated hRSV release at 48 hpi, compared with controls (Fig. 8*A* and 8*B*). This effect was dependent on the Ab dilution and the time of infection. eGPF intensity and percentage of coverage are surrogates for RSV gene transcription kinetics whereas virus TCID_50_ titrations measure replication-competent virus release, and virus transcription evidently occurs before assembly and release. Therefore, peak effects of anti-CEACAM1 treatment on RSV replication at 24 hpi (eGFP intensities and coverage) and at 48 hpi (TCID_50_ titrations) is consistent with the relative kinetics of gene transcription and virus release These data are consistent with an antiviral activity of CEACAM1 against hRSV.

##### Protein Content in NPAs from hRSV-infected Infants Reflects the WD-PBECs Data

**Fig. 9.**
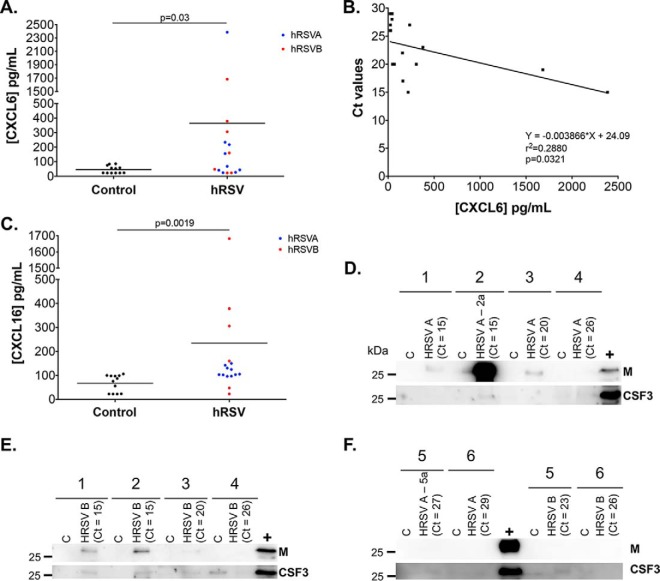
**Detection of host and viral proteins in NPAs from hRSV-positive children.** NPA samples from hRSVA-positive (*n* = 6) and hRSVB-positive (*n* = 6) infants were analyzed by ELISA and Western blotting and compared with non-infected control. Samples were collected and prepared as per methods. CXCL6 quantities (*A*) were measured by ELISA, and correlation between CXCL6 concentration and viral load (Ct values) was calculated by linear regression analysis (*B*). CXCL16 quantities (*C*) were also measured by ELISA. Eight μg protein/lane were analyzed by immunoblotting with antibodies against hRSV M protein and CSF3. Patient numbers are indicated above (See supplemental Table S10) (*D*, *E*, *F*). Whole cell lysate from hRSV A2-infected HEp-2 cells was used as a positive control (+). Ct values from the diagnostic tests are indicated. Statistical significance was established using a Mann-Whitney test.

To determine whether CXCL6, CXCL16 and CSF3 induction following hRSV infection in WD-PBECs corresponded with hRSV-infected infants, NPAs from hRSV-positive and uninfected infants (supplemental Table S10) were tested by ELISA and immunoblotting. NPA use was dictated by appropriate sample availability. However, although the WD-PBEC cultures were derived from tracheobronchial cells, NPA clinical samples were considered appropriate, as evidence suggests that nasal epithelium is an appropriate surrogate for bronchial epithelium in relation to hRSV infection (17). The concentrations of CXCL6 (Fig. 9*A*) and CXCL16 (Fig. 9*C*) were significantly increased in NPAs from hRSV-positive infants. There was a small but significant correlation between CXCL6 concentrations and viral load (Ct values) in NPAs (Fig. 9*B*), suggesting that the release of CXCL6 might be dependent on the kinetic of viral replication. Similarly, CSF3 was detected by Western blotting in most samples from sick children compare with controls (Fig. 9*D*–9*F*). Furthermore, hRSV matrix protein was detected by Western blotting only in hRSV-positive samples with Ct values <20 cycles.

## DISCUSSION

The proteome of airway secretions was unknown in the context of an acute respiratory virus infections. This study determined the impact of hRSV on the protein composition of airway epithelium secretions. For this purpose, WD-PBECs were infected with a clinical isolate of hRSV (BT2a), or mock-infected, and the apical and basolateral secretomes were profiled using quantitative proteomics.

The data indicated that the directional nature of the airway secretome at homeostasis (5) was markedly disrupted by hRSV. This was primarily evidenced by the altered polarity of secretion of a large panel of proteins (Fig. 4), encompassing multiple biological processes, and was indicative of a potentially dysfunctional epithelial barrier. Indeed, such epithelial leakage resembles the increased paracellular permeability observed in HAE cultures originating from asthmatic patients (51) or in polarized monolayers with CF defects (52). In those models, disruption of epithelial tight junction integrity was evident, and was associated with decreased transepithelial electrical resistance (TEER) and increased permeability to fluorescently labeled macromolecules. Interestingly, these effects were also noted after hRSV infection in polarized respiratory epithelial cell monolayers (53), and bronchial, but not nasal, HAE cultures (17, 18). One consequence of a “leaky” epithelium, in the HAE model at least, was to exacerbate an otherwise compartmentalized inflammatory response (54, 55), by increasing the exposure of sub-epithelial tissues to luminal antigens (allergens and immunogenic ligands).

Furthermore, disordered immune responses is consistent with the exacerbated inflammation of the bronchiolar and alveolar regions observed in severe hRSV-associated pathologies (reviewed in (56)). hRSV-associated inflammation is thought to be Th2-mediated and characterized by a large infiltration of neutrophils in the airways (44), consistent with elevated IL-6, CXCL8, and CXCL10 responses (15, 45, 46). Altered CXCL6, CSF3, SECTM1, and CXCL16 secretions were, therefore, highly relevant in this context considering their functions as modulators of neutrophil and T cell activity (Fig. 5). Conversely, the loss of MUC13 secretion following infection was intriguing because it is a transmembrane mucosal protein with pro-inflammatory properties (57). CSF3 is a neutrophil growth factor, whereas CXCL6 is chemotactic for neutrophils and binds the same receptors as CXCL8 (CXCR1/CXCR2) (58). SECTM1 is a membrane/secreted airway epithelium-derived protein that was shown to sustain an amplifying loop of neutrophilic inflammation during pneumococcal pneumonia (43). CXCL16 is an epithelium-derived chemokine that recruits and activates lung T cells via the CXCR6 receptor (59). CXCL6 and CXCL16 were particularly noteworthy because of their increased concentrations in NPAs from hRSV-positive children (Fig. 9*A* and 9*C*) and their association with several chronic lung pathologies, such as idiopathic pulmonary fibrosis (IPF), CF, chronic rhinosinusitis (CRS) and asthma. In CF and CRS patients, CXCL6 was detected in lung epithelium goblet cells and was a more potent neutrophil chemoattractant than CXCL8, whereas treatment with an anti-CXCL6 antibody reduced neutrophil influx and inflammation in a mouse model of IPF (59–63). Similarly, CXCR6 was expressed on lung-infiltrating Th2 cells in a mouse model of asthma (62) and in asthmatic patients (61). In addition, whereas MUC13 exacerbated the responsiveness of intestinal epithelial cell lines to multiple TLR ligands, leading to increased IL-8 and TNF-α production (57), its expression was only up-regulated in NPAs from patients with severe hRSV-associated disease, in contrast to milder cases (64). Overall, our data suggest that CXCL6, CXCL16, CSF3 and, to a lesser extent, SECTM1 and MUC13, may constitute important but previously unidentified drivers/modulators of neutrophil infiltration and recruitment and/or activation of Th2 cells to the lungs during acute hRSV infection.

In addition to identifying novel hRSV-induced mediators associated with recruitment of immunopathogenic immune cells, this work also highlighted direct cytophysiological and antiviral responses of airway epithelium to hRSV infection. For example, BST2 and CEACAM1, which have known antiviral properties against HIV, influenza virus or hMPV, were specifically induced upon hRSV infection (37, 38, 42) and in the case of CEACAM1 this correlated with previous data in A549 and undifferentiated primary bronchial epithelial cells (49). Importantly, this is the first description of the involvement of CEACAM1 in anti-hRSV defense (Fig. 8). Like BST2, CEACAM1 is normally a membrane-associated protein, but its secretion may be explained by soluble isoforms of the protein (48). Active cleavage of native CEACAM1 from cell surfaces is also possible, especially in such a protease-rich environment. In this study, CEACAM1 was identified as a novel secreted factor with antiviral properties against hRSV in the epithelium environment. Another novel observation was the loss of detection of two membrane transporters, SLC2A2 and ATPV6E6, which are involved in maintaining cation/Ca^2+^ homeostasis (Fig. 5*A* and supplemental Table S8). Indeed, efficient mucociliary clearance relies essentially on coordinated cilia movement, which is partly controlled by tight regulation of intracellular and extracellular levels of Ca^2+^ (65, 66). Altering the expression/secretion of these host factors suggests that hRSV might affect Ca^2+^ levels, which in turn could lead to dysregulation of cilia beating. This is consistent with the ability of hRSV to induce cilia beating dyskinesia, at least in the HAE model (18). A reduced mucociliary clearance may indeed exacerbate hRSV-associated pathologies because it drives the transport of inhaled particles and pathogens trapped in the mucus out of the airways and toward the oropharynx.

In conclusion, global analysis of the apical and basolateral secretomes following RSV infection revealed novel interactions between hRSV and the airway epithelium. It also identified novel anti-hRSV innate immune responses with potential for therapeutic intervention. The application of proteomics to our hRSV/WD-PBEC model, therefore, provided extensive new insights into hRSV/host interactions and the rationale for therapeutic target discovery.

## DATA AVAILABILITY

The mass spectrometry proteomics data have been deposited to the ProteomeXchange Consortium via the PRIDE (28) partner repository with the data set identifier PXD013661. Data can be accessed at https://www.ebi.ac.uk/pride/.

## Supplementary Material

supplemental Fig. S5

Tables S1 to S10

Supplementary figures S1 to S9

## Footnotes

^1^ The abbreviations used are:

COPD: chronic obstructive pulmonary disease
RSV: respiratory syncytial virus
HAE: human airway epithelial model
WD-PBECs: well-differentiated pediatric bronchial epithelial cells
BALF: bronchoalveolar lavage fluid
NPA: nasopharyngeal aspirate
CF: cystic fibrosis
ALRI: airway lower respiratory tract infection.

## References

[1] Kesimer M., Kirkham S., Pickles R. J., Henderson A. G., Alexis N. E., DeMaria G., Knight D., Thornton D. J., and Sheehan J. K. (2009) Tracheobronchial air-liquid interface cell culture: a model for innate mucosal defense of the upper airways? Am. J. Physiol. Cell. Mol. Physiol. 296, L92–L100 10.1152/ajplung.90388.2008 PMC2636953 18931053

[2] Kesimer M., Scull M., Brighton B., DeMaria G., Burns K., O'Neal W., Pickles R. J., and Sheehan J. K. (2009) Characterization of exosome-like vesicles released from human tracheobronchial ciliated epithelium: a possible role in innate defense. FASEB J. 23, 1858–1868 19190083 10.1096/fj.08-119131 PMC2698655

[3] Ali M., Lillehoj E. P., Park Y., Kyo Y., and Kim K. C. (2011) Analysis of the proteome of human airway epithelial secretions. Proteome Sci. 9, 4 21251289 10.1186/1477-5956-9-4 PMC3036598

[4] Kesimer M., Ehre C., Burns K. A., Davis C. W., Sheehan J. K., and Pickles R. J. (2013) Molecular organization of the mucins and glycocalyx underlying mucus transport over mucosal surfaces of the airways. Mucosal Immunol. 6, 379–392 22929560 10.1038/mi.2012.81 PMC3637662

[5] Pillai D. K., Sankoorikal B. J. V., Johnson E., Seneviratne A. N., Zurko J., Brown K. J., Hathout Y., and Rose M. C. (2014) Directional secretomes reflect polarity-specific functions in an in vitro model of human bronchial epithelium. Am. J. Respir. Cell Mol. Biol. 50, 292–300 24010916 10.1165/rcmb.2013-0188OC PMC3930950

[6] Tu C., Jacob Mammen M., Li J., Shen X., Jiang X., Hu Q., Wang J., Sethi S., and Qu J. (2014) Large-scale, ion-current-based proteomics investigation of bronchoalveolar lavage fluid in chronic obstructive pulmonary disease patients. J. Proteome Res. 13, 627–639 24188068 10.1021/pr4007602 PMC4073647

[7] Wu J., Kobayashi M., Sousa E. A., Liu W., Cai J., Goldman S. J., Dorner A. J., Projan S. J., Kavuru M. S., Qiu Y., and Thomassen M. J. (2005) Differential proteomic analysis of bronchoalveolar lavage fluid in asthmatics following segmental antigen challenge. Mol. Cell. Proteomics 4, 1251–1264 15951573 10.1074/mcp.M500041-MCP200

[8] MacGregor G., Gray R. D., Hilliard T. N., Imrie M., Boyd A. C., Alton E. W. F. W., Bush A., Davies J. C., Innes J. A., Porteous D. J., and Greening A. P. (2008) Biomarkers for cystic fibrosis lung disease: Application of SELDI-TOF mass spectrometry to BAL fluid. J. Cyst. Fibros. 7, 352–358 18243068 10.1016/j.jcf.2007.12.005

[9] Gharib S. A., Vaisar T., Aitken M. L., Park D. R., Heinecke J. W., and Fu X. (2009) Mapping the lung proteome in cystic fibrosis. J. Proteome Res. 8, 3020–3028 19354268 10.1021/pr900093j

[10] Nguyen E. V., Gharib S. A., Crothers K., Chow Y. H., Park D. R., Goodlett D. R., and Schnapp L. M. (2014) Proteomic landscape of bronchoalveolar lavage fluid in human immunodeficiency virus infection. Am. J. Physiol. Lung Cell Mol. Physiol 306, L35–L42 24213920 10.1152/ajplung.00140.2013 PMC3920210

[11] Fornander L., Ghafouri B., Kihlström E., Åkerlind, Schön B. T., Tagesson C., and Lindahl M. (2011) Innate immunity proteins and a new truncated form of SPLUNC1 in nasopharyngeal aspirates from infants with respiratory syncytial virus infection. Proteomics Clin. Appl. 5, 513–522 21805676 10.1002/prca.201100016

[12] Nair H., Nokes D. J., Gessner B. D., Dherani M., Madhi S. A., Singleton R. J., O'Brien K. L., Roca A., Wright P. F., Bruce N., Chandran A., Theodoratou E., Sutanto A., Sedyaningsih E. R., Ngama M., Munywoki P. K., Kartasasmita C., Simões E. A., Rudan I., Weber M. W., and Campbell H. (2010) Global burden of acute lower respiratory infections due to respiratory syncytial virus in young children: a systematic review and meta-analysis. Lancet 375, 1545–1555 20399493 10.1016/S0140-6736(10)60206-1 PMC2864404

[13] Zhang L., Peeples M. E., Boucher R. C., Collins P. L., and Pickles R. J. (2002) Respiratory syncytial virus infection of human airway epithelial cells is polarized, specific to ciliated cells, and without obvious cytopathology. J. Virol. 76, 5654–5666 11991994 10.1128/JVI.76.11.5654-5666.2002 PMC137037

[14] Liesman R. M., Buchholz U. J., Luongo C. L., Yang L., Proia A. D., DeVincenzo J. P., Collins P. L., and Pickles R. J. (2014) RSV-encoded NS2 promotes epithelial cell shedding and distal airway obstruction. J. Clin. Invest. 124, 2219–2233 24713657 10.1172/JCI72948 PMC4001550

[15] Villenave R., Thavagnanam S., Sarlang S., Parker J., Douglas I., Skibinski G., Heaney L. G., McKaigue J. P., Coyle P. V., Shields M. D., and Power U. F. (2012) In vitro modeling of respiratory syncytial virus infection of pediatric bronchial epithelium, the primary target of infection in vivo. Proc. Natl. Acad. Sci. 109, 5040–5045 22411804 10.1073/pnas.1110203109 PMC3323997

[16] Mata M., Sarrion I., Armengot M., Carda C., Martinez I., Melero J. A., and Cortijo J. (2012) Respiratory syncytial virus inhibits ciliagenesis in differentiated normal human bronchial epithelial cells: effectiveness of N-acetylcysteine. PLoS ONE 7, e48037 23118923 10.1371/journal.pone.0048037 PMC3485262

[17] Guo-Parke H., Canning P., Douglas I., Villenave R., Heaney L. G., Coyle P. V., Lyons J. D., Shields M. D., and Power U. F. (2013) Relative respiratory syncytial virus cytopathogenesis in upper and lower respiratory tract epithelium. Am. J. Respir. Crit. Care Med. 188, 842–851 23952745 10.1164/rccm.201304-0750OC

[18] Smith C. M., Kulkarni H., Radhakrishnan P., Rutman A., Bankart M. J., Williams G., Hirst R. A., Easton A. J., Andrew P. W., and O'Callaghan C. (2014) Ciliary dyskinesia is an early feature of respiratory syncytial virus infection. Eur. Respir. J. 43, 485–496 23520320 10.1183/09031936.00205312

[19] Jumat M. R., Yan Y., Ravi L. I., Wong P., Huong T. N., Li C., Tan B. H., Wang D. Y., and Sugrue R. J. (2015) Morphogenesis of respiratory syncytial virus in human primary nasal ciliated epithelial cells occurs at surface membrane microdomains that are distinct from cilia. Virology 484, 395–411 26231613 10.1016/j.virol.2015.05.014

[20] Broadbent L., Villenave R., Guo-Parke H., Douglas I., Shields M. D., and Power U. F. (2016) in Methods in Molecular Biology, ed Tripp R. A. and Jorquera P. A., pp 119–139, Springer Nature, New York, NY 10.1007/978-1-4939-3687-8_9 27464691

[21] Villenave R., Shields M. D., and Power U. F. (2013) Respiratory syncytial virus interaction with human airway epithelium. Trends Microbiol. 21, 238–244 23523320 10.1016/j.tim.2013.02.004

[22] Villenave R., O'Donoghue D., Thavagnanam S., Touzelet O., Skibinski G., Heaney L. G., McKaigue J. P., Coyle P. V., Shields M. D., and Power U. F. (2011) Differential cytopathogenesis of respiratory syncytial virus prototypic and clinical isolates in primary pediatric bronchial epithelial cells. Virol. J. 8, 43 21272337 10.1186/1743-422X-8-43 PMC3039598

[23] Dougherty R. (1964) in Techniques in Experimental Virology, ed Harris R, pp 183–186, Academic Press, New York, NY

[24] García-Dorival I., Wu W., Dowall S., Armstrong S., Touzelet O., Wastling J., Barr J. N., Matthews D., Carroll M., Hewson R., and Hiscox J. A. (2014) Elucidation of the ebola virus VP24 cellular interactome and disruption of virus biology through targeted inhibition of host-cell protein function. J. Proteome Res. 13, 5120–5135 25158218 10.1021/pr500556d

[25] Cox J., and Mann M. MaxQuant enables high peptide identification rates, individualized p.p.b.-range mass accuracies and proteome-wide protein quantification. Nat. Biotechnol. 26, 1367–1372 10.1038/nbt.1511 19029910

[26] Cox J., Neuhauser N., Michalski A., Scheltema R. A., Olsen J. V., and Mann M. (2011) Andromeda: A peptide search engine integrated into the MaxQuant environment. J. Proteome Res. 10, 1794–1805 21254760 10.1021/pr101065j

[27] Apweiler R., Bateman A., Martin M. J., O'Donovan C., Magrane M., Alam-Faruque Y., Alpi E., Antunes R., Arganiska J., Casanova E. B., Bely B., Bingley M., Bonilla C., Britto R., Bursteinas B., Chan W. M., Chavali G., Cibrian-Uhalte E., Da Silva A., De Giorgi M., Fazzini F., Gane P., Castro L. G., Garmiri P., Hatton-Ellis E., Hieta R., Huntley R., Legge D., Liu W., Luo J., MacDougall A., Mutowo P., Nightingale A., Orchard S., Pichler K., Poggioli D., Pundir S., Pureza L., Qi G., Rosanoff S., Sawford T., Shypitsyna A., Turner E., Volynkin V., Wardell T., Watkins X., Corbett M., Donnelly M., Van Rensburg P., Goujon M., McWilliam H., Lopez R., Xenarios I., Bougueleret L., Bridge A., Poux S., Redaschi N., Aimo L., Auchincloss A., Axelsen K., Bansal P., Baratin D., Binz P. A., Blatter M. C., Boeckmann B., Bolleman J., Boutet E., Breuza L., Casal-Casas C., De Castro E., Cerutti L., Coudert E., Cuche B., Doche M., Dornevil D., Duvaud S., Estreicher A., Famiglietti L., Feuermann M., Gasteiger E., Gehant S., Gerritsen V., Gos A., Gruaz-Gumowski N., Hinz U., Hulo C., James J., Jungo F., Keller G., Lara V., Lemercier P., Lew J., Lieberherr D., Lombardot T., Martin X., Masson P., Morgat A., Neto T., Paesano S., Pedruzzi I., Pilbout S., Pozzato M., Pruess M., Rivoire C., Roechert B., Schneider M., Sigrist C., Sonesson K., Staehli S., Stutz A., Sundaram S., Tognolli M., Verbregue L., Veuthey A. L., Wu C. H., Arighi C. N., Arminski L., Chen C., Chen Y., Garavelli J. S., Huang H., Laiho K., McGarvey P., Natale D. A., Suzek B. E., Vinayaka C. R., Wang Q., Wang Y., Yeh L. S., Yerramalla M. S., and Zhang J. (2014) Activities at the Universal Protein Resource (UniProt). Nucleic Acids Res. 42, D191–D198 24253303 10.1093/nar/gkt1140 PMC3965022

[28] Perez-Riverol Y., Csordas A., Bai J., Bernal-Llinares M., Hewapathirana S., Kundu D. J., Inuganti A., Griss J., Mayer G., Eisenacher M., Pérez E., Uszkoreit J., Pfeuffer J., Sachsenberg T., Yilmaz Ş Tiwary S., Cox J., Audain E., Walzer M., Jarnuczak A. F., Ternent T., Brazma A., and Vizcaíno J. A. (2019) The PRIDE database and related tools and resources in 2019: Improving support for quantification data. Nucleic Acids Res. 47, D442–D450 30395289 10.1093/nar/gky1106 PMC6323896

[29] Uhlen M., Fagerberg L., Hallstrom B. M., Lindskog C., Oksvold P., Mardinoglu A., Sivertsson A., Kampf C., Sjostedt E., Asplund A., Olsson I., Edlund K., Lundberg E., Navani S., Szigyarto C. A.-K., Odeberg J., Djureinovic D., Takanen J. O., Hober S., Alm T., Edqvist P.-H., Berling H., Tegel H., Mulder J., Rockberg J., Nilsson P., Schwenk J. M., Hamsten M., von Feilitzen K., Forsberg M., Persson L., Johansson F., Zwahlen M., von Heijne G., Nielsen J., and Ponten F. (2015) Tissue-based map of the human proteome. Science 347, 1260419–1260419 25613900 10.1126/science.1260419

[30] Petersen T. N., Brunak S., Von Heijne G., and Nielsen H. (2011) SignalP 4.0: Discriminating signal peptides from transmembrane regions. Nat. Methods 8, 785–786 21959131 10.1038/nmeth.1701

[31] Käll L., Krogh A., and Sonnhammer E. L. L. (2007) Advantages of combined transmembrane topology and signal peptide prediction-the Phobius web server. Nucleic Acids Res. 35, 429–432 10.1093/nar/gkm256 PMC1933244 17483518

[32] Viklund H., Bernsel A., Skwark M., and Elofsson A. (2008) SPOCTOPUS: A combined predictor of signal peptides and membrane protein topology. Bioinformatics 24, 2928–2929 18945683 10.1093/bioinformatics/btn550

[33] Krogh a. Larsson B., von Heijne G., and Sonnhammer E. L. (2001) Predicting transmembrane protein topology with a hidden Markov model: application to complete genomes. J. Mol. Biol. 305, 567–580 11152613 10.1006/jmbi.2000.4315

[34] Bendtsen J. D., Jensen L. J., Blom N., Von Heijne G., and Brunak S. (2004) Feature-based prediction of non-classical and leaderless protein secretion. Protein Eng. Des. Sel. 17, 349–356 15115854 10.1093/protein/gzh037

[35] Emanuelsson O., Brunak S., von Heijne G., and Nielsen H. (2007) Locating proteins in the cell using TargetP, SignalP and related tools. Nat. Protoc. 2, 953–971 17446895 10.1038/nprot.2007.131

[36] Keerthikumar S., Chisanga D., Ariyaratne D., Al Saffar H., Anand S., Zhao K., Samuel M., Pathan M., Jois M., Chilamkurti N., Gangoda L., and Mathivanan S. (2016) ExoCarta: a web-based compendium of exosomal cargo. J. Mol. Biol. 428, 688–692 26434508 10.1016/j.jmb.2015.09.019 PMC4783248

[37] Vitenshtein A., Weisblum Y., Hauka S., Halenius A., Oiknine-Djian E., Tsukerman P., Bauman Y., Bar-On Y., Stern-Ginossar N., Enk J., Ortenberg R., Tai J., Markel G., Blumberg R. S., Hengel H., Jonjic S., Wolf D. G., Adler H., Kammerer R., and Mandelboim O. (2016) CEACAM1-mediated inhibition of virus production. Cell Rep. 15, 2331–2339 27264178 10.1016/j.celrep.2016.05.036 PMC4914772

[38] Diab M., Vitenshtein A., Drori Y., Yamin R., Danziger O., Zamostiano R., Mandelboim M., Bacharach E., Mandelboim O., Diab M., Vitenshtein A., Drori Y., Yamin R., Danziger O., Zamostiano R., Mandelboim M., Bacharach E., and Mandelboim O. (2014) Suppression of human metapneumovirus (HMPV) infection by the innate sensing gene CEACAM1. Oncotarget 7, 66468–66479 10.18632/oncotarget.11979 PMC5341814 27634893

[39] Baxter A., Thain S., Banerjee A., Haswell L., Parmar A., Phillips G., and Minet E. (2015) Targeted omics analyses, and metabolic enzyme activity assays demonstrate maintenance of key mucociliary characteristics in long term cultures of reconstituted human airway epithelia. Toxicol. Vitr. 29, 864–875 10.1016/j.tiv.2015.03.004 25863282

[40] Zhao Y., Jamaluddin M., Zhang Y., Sun H., Ivanciuc T., Garofalo R. P., and Brasier A. R. (2017) Systematic analysis of cell-type differences in the epithelial secretome reveals insights into the pathogenesis of respiratory syncytial virus–induced lower respiratory tract infections. J. Immunol. 198, 3345–3364 28258195 10.4049/jimmunol.1601291 PMC5380581

[41] Day C., Patel R., Guillen C., and Wardlaw A. J. (2009) The chemokine cxcl16 is highly and constitutively expressed by human bronchial epithelial cells. Exp. Lung Res. 35, 272–283 19415545 10.1080/01902140802635517 PMC2685639

[42] Mangeat B., Cavagliotti L., Lehmann M., Gers-Huber G., Kaur I., Thomas Y., Kaiser L., and Piguet V. (2012) Influenza virus partially counteracts restriction imposed by tetherin/BST-2. J. Biol. Chem. 287, 22015–22029 22493439 10.1074/jbc.M111.319996 PMC3381161

[43] Hirofumi Kamata Kazuko Yamamoto Gregory Wasserman A., Mary Zabinski C., Constance Yuen K., Wing Yi Lung Adam Gower C., Anna Belkina C., Maria Ramirez I., Jane Deng C., Lee Quinton J., Matthew Jones R., JPM. (2016) Epithelial cell–derived secreted and transmembrane 1a signals to activated neutrophils during pneumococcal pneumonia. Am. J. Respir. Cell Mol. Biol. 55, 407–418 27064756 10.1165/rcmb.2015-0261OC PMC5023025

[44] Mcnamara P. S., Ritson P., Selby A., Hart C. A., and Smyth R. L. (2003) Bronchoalveolar lavage cellularity in infants with severe respiratory syncytial virus bronchiolitis. Arch Dis Child 88, 922–926 14500316 10.1136/adc.88.10.922 PMC1719332

[45] Juntti H., Osterlund P., Kokkonen J., Dunder T., Renko M., Pokka T., Julkunen I., and Uhari M. (2009) Cytokine responses in cord blood predict the severity of later respiratory syncytial virus infection. J. Allergy Clin. Immunol 124, 52 19482350 10.1016/j.jaci.2009.04.014

[46] McNamara P. S., Flanagan B. F., Hart C. A. S. R. (2005) Production of chemokines in the lungs of infants with severe respiratory syncytial virus bronchiolitis. J. Infect Dis. 191, 1225–1232 15776367 10.1086/428855

[47] Nguyen E. V., Gharib S. A., Schnapp L. M., and Goodlett D. R. (2014) Shotgun MS proteomic analysis of bronchoalveolar lavage fluid in normal subjects. Proteomics - Clin. Appl. 8, 737–747 24616423 10.1002/prca.201300018 PMC4239657

[48] Terahara K., Yoshida M., Taguchi F., Igarashi O., Nochi T., Gotoh Y., Yamamoto T., Tsunetsugu-Yokota Y., Beauchemin N., and Kiyono H. (2009) Expression of newly identified secretory CEACAM1a isoforms in the intestinal epithelium. Biochem. Biophys. Res. Commun. 383, 340–346 19358828 10.1016/j.bbrc.2009.04.008

[49] Avadhanula V., Rodriguez C. A., Devincenzo J. P., Wang Y., Webby R. J., Ulett G. C., and Adderson E. E. (2006) Respiratory viruses augment the adhesion of bacterial pathogens to respiratory epithelium in a viral species- and cell type-dependent manner. J. Virol. 80, 1629–1636 16439519 10.1128/JVI.80.4.1629-1636.2006 PMC1367158

[50] Hillyer P., Shepard R., Uehling M., Krenz M., Sheikh F., Thayer K. R., Huang L., Yan L., Panda D., Luongo C., Buchholz U. J., Collins P. L., Donnelly R. P., and Rabin R. L. (2018) Differential responses by human respiratory epithelial cell lines to respiratory syncytial virus reflect distinct patterns of infection control. J. Virol. 92, 1–21 10.1128/JVI.02202-17 PMC6052282 29769339

[51] Xiao C., Puddicombe S. M., Field S., Haywood J., Broughton-Head V., Puxeddu I., Haitchi H. M., Vernon-Wilson E., Sammut D., Bedke N., Cremin C., Sones J., Djukanović R., Howarth P. H., Collins J. E., Holgate S. T., Monk P., and Davies D. E. (2011) Defective epithelial barrier function in asthma. J. Allergy Clin. Immunol. 128, 549–556 21752437 10.1016/j.jaci.2011.05.038

[52] Weiser N., Molenda N., Urbanova K., Bähler M., Pieper U., Oberleithner H., and Schillers H. (2011) Paracellular permeability of bronchial epithelium is controlled by CFTR. Cell Physiol. Biochem. 28, 289–296 21865736 10.1159/000331742

[53] Rezaee F., DeSando Sa Ivanov A. I., Chapman T. J., Knowlden S. A., Beck L. A., and Georas S. N. (2013) Sustained protein kinase D activation mediates respiratory syncytial virus-induced airway barrier disruption. J. Virol. 87, 11088–11095 23926335 10.1128/JVI.01573-13 PMC3807305

[54] Humlicek A. L., Manzel L. J., Chin C. L., Shi L., Excoffon KJDa Winter M. C., Shasby D. M., and Look D. C. (2014) Paracellular permeability restricts airway epithelial responses to selectively allow activation by mediators at the basolateral surface. J. Immunol. 178, 6395–6403 10.4049/jimmunol.178.10.6395 17475869

[55] Gustavo N., Shehlanoor H., Geovanny F. P., Krishna P., Humaira M., Aleeza A., Justin W., Stephen E., Anamaris M. C. P., Dinesh K. P., and Mary C. R. (2014) Directional secretory response of double stranded RNA-induced thymic stromal lymphopoetin (TSLP) and CCL11/eotaxin-1 in human asthmatic airways. PLoS ONE 9, e115398 25546419 10.1371/journal.pone.0115398 PMC4278901

[56] Touzelet O., and Power U. F. (2011) in HUMAN RESPIRATORY SYNCYTIAL VIRUS INFECTION, ed Resh B, InTech, Rijeka, Croatia, pp 97–122

[57] Sheng Y. H., Triyana S., Wang R., Das I., Gerloff K., Florin T. H., Sutton P., and Mcguckin M. A. (2012) MUC1 and MUC13 differentially regulate epithelial inflammation in response to inflammatory and infectious stimuli. Mucosal Immunol. 6, 557–568 23149663 10.1038/mi.2012.98

[58] Wuyts A., Van Osselaer N., Haelens A., Samson I., Herdewijn P., Ben-Baruch A., Oppenheim J. J., Proost P., and Van Damme J. (1997) Characterization of synthetic human granulocyte chemotactic protein 2: Usage of chemokine receptors CXCR1 and CXCR2 and in vivo inflammatory properties. Biochemistry 36, 2716–2723 9054580 10.1021/bi961999z

[59] Jovic S., Linge H. M., Shikhagaie M. M., Olin A. I., Lannefors L., Erjefält J. S., Mörgelin M., and Egesten A. (2016) The neutrophil-recruiting chemokine GCP-2/CXCL6 is expressed in cystic fibrosis airways and retains its functional properties after binding to extracellular DNA. Mucosal Immunol. 9, 112–123 25993443 10.1038/mi.2015.43

[60] Rudack C., Sachse F., and Alberty J. (2006) Primary role of growth-related oncogene-?? and granulocyte chemotactic protein-2 as neutrophil chemoattractants in chronic rhinosinusitis. Clin. Exp. Allergy 36, 748–759 16776676 10.1111/j.1365-2222.2006.02501.x

[61] Besnard A.-G., Struyf S., Guabiraba R., Fauconnier L., Rouxel N., Proost P., Uyttenhove C., Snick Van J., Couillin I., and Ryffel B. (2013) CXCL6 antibody neutralization prevents lung inflammation and fibrosis in mice in the bleomycin model. J. Leukoc. Biol 4, 1317–1323 10.1189/jlb.0313140 23975892

[62] Latta M., Mohan K., and Issekutz T. B. (2007) CXCR6 is expressed on T cells in both T helper type 1 (Th1) inflammation and allergen-induced Th2 lung inflammation but is only a weak mediator of chemotaxis. Immunology 121, 555–564 17437534 10.1111/j.1365-2567.2007.02603.x PMC2265962

[63] Morgan A. J., Guillen C., Symon F. A., Huynh T. T., Berry M. A., Entwisle J. J., Briskin M., Pavord I. D., and Wardlaw A. J. (2005) Expression of CXCR6 and its ligand CXCL16 in the lung in health and disease. Clin. Exp. Allergy 35, 1572–1580 16393323 10.1111/j.1365-2222.2005.02383.x

[64] Van Den Kieboom C. H., Ahout I. M. L., Zomer A., Brand K. H., De Groot R., Ferwerda G., and De Jonge M. I. (2015) Nasopharyngeal gene expression, a novel approach to study the course of respiratory syncytial virus infection. Eur. Respir. J. 45, 718–725 25261323 10.1183/09031936.00085614

[65] Schmid A., and Salathe M. (2011) Ciliary beat co-ordination by calcium. Biol. Cell 103, 159–169 21401526 10.1042/BC20100120

[66] Droguett K., Rios M., Carreño D. V., Navarrete C., Fuentes C., Villalón M., and Barrera N. P. (2017) An autocrine ATP release mechanism regulates basal ciliary activity in airway epithelium. J. Physiol. 595, 4755–4767 28422293 10.1113/JP273996 PMC5509870

