# Supplementary material for: The Secretome Profiling of a Pediatric Airway Epithelium Infected with hRSV Identified Aberrant Apical/Basolateral Trafficking and Novel Immune Modulating (CXCL6, CXCL16, CSF3) and Antiviral (CEACAM1) Proteins
Source: Mol Cell Proteomics. 2020 Feb 19;19(5):793–807. doi: 10.1074/mcp.RA119.001546 (PMC7196588; doi:10.1074/mcp.RA119.001546)
Supplement: supplemental Fig. S5 [file RA119.001546_index.html]

Supplement to The secretome profiling of a pediatric airway epithelium infected with hRSV identified aberrant apical/basolateral trafficking and novel immune modulating (CXCL6, CXCL16, CSF3) and antiviral (CEACAM1) proteins. | Molecular & Cellular Proteomics

## Supplemental Data

- Tables S1 to S10 - Table S1: Search parameters and acceptance criteria; Table S2: Summary information for all the raw files processed with a single MaxQuant run; Table S3: List of all the proteins identified (raw data); Table S4: List of all the peptides identified (raw data); Table S5: Protein list after filtering; Table S6: Gene ontology biological process (GOBP) enrichment analysis for Mock unique and enriched proteins; Table S7: Mock enriched GOBP potentially affected by hRSV; Table S8: Gene ontology biological process (GOBP) enrichment analysis for hRSV-induced proteins; Table S9: Reference list of human proteins previously detected in clinical samples and HAE cells; Table S10: Ct values of NPAs from hRSVA- and hRSVB-positive children, and matched controls;
- Supplementary figures S1 to S9 - Supplementary figures S1 to S9
