# Supplementary material for: The Secretome Profiling of a Pediatric Airway Epithelium Infected with hRSV Identified Aberrant Apical/Basolateral Trafficking and Novel Immune Modulating (CXCL6, CXCL16, CSF3) and Antiviral (CEACAM1) Proteins
Source: Mol Cell Proteomics. 2020 Feb 19;19(5):793–807. doi: 10.1074/mcp.RA119.001546 (PMC7196588; doi:10.1074/mcp.RA119.001546)
Supplement: Supplementary figures S1 to S9 [file 152619_2_supp_473972_q5prpt.pdf]

SUPPLEMENTARY FIGURES S1 TO S9

Title: The secretome profiling of a pediatric airway epithelium infected with human respiratory syncytial virus (hRSV) identified aberrant apical/basolateral trafficking and novel immune modulating (CXCL6, CXCL16, CSF3) and antiviral (CEACAM1) proteins.

Olivier Touzelet, Lindsay Broadbent, Stuart D. Armstrong, Waleed Aljabr, Elaine Cloutman-Green, Ultan F. Power and Julian A. Hiscox

**S1**	Individual channel for immunofluorescence staining of WD-PBECs infected with hRSV BT2a (figure 1B).
**S2**	Virus growth kinetics in individual donor and distribution of cells.
**S3**	Decision tree leading to the classification of host proteins between secreted via a signal peptide, secreted in a non-classical pathway, intracellular or membrane.
**S4**	Apical and basolateral distribution of each protein category based on the protein number and abundance.
**S5**	Proteins differentially abundant in the apical and basolateral compartments of mock and hRSV BT2a-infected WD-PBEC cultures.
**S6**	Hierarchical clustering of proteins whose abundance was significantly different between the apical and the basolateral side of (A) mock- and (B) hRSV-infected cultures.
**S7**	Enriched BP in mock apical and basolateral secretomes.
**S8**	Apical/basolateral protein abundance ratios in the secretome of control and RSV-infected WD-PBECs cultures.
**S9**	eGFP intensity in BEAS-2B cells infected with hRSV/eGFP and incubated with a blocking antibody anti-CEACAM1.

Figure S1 bis:

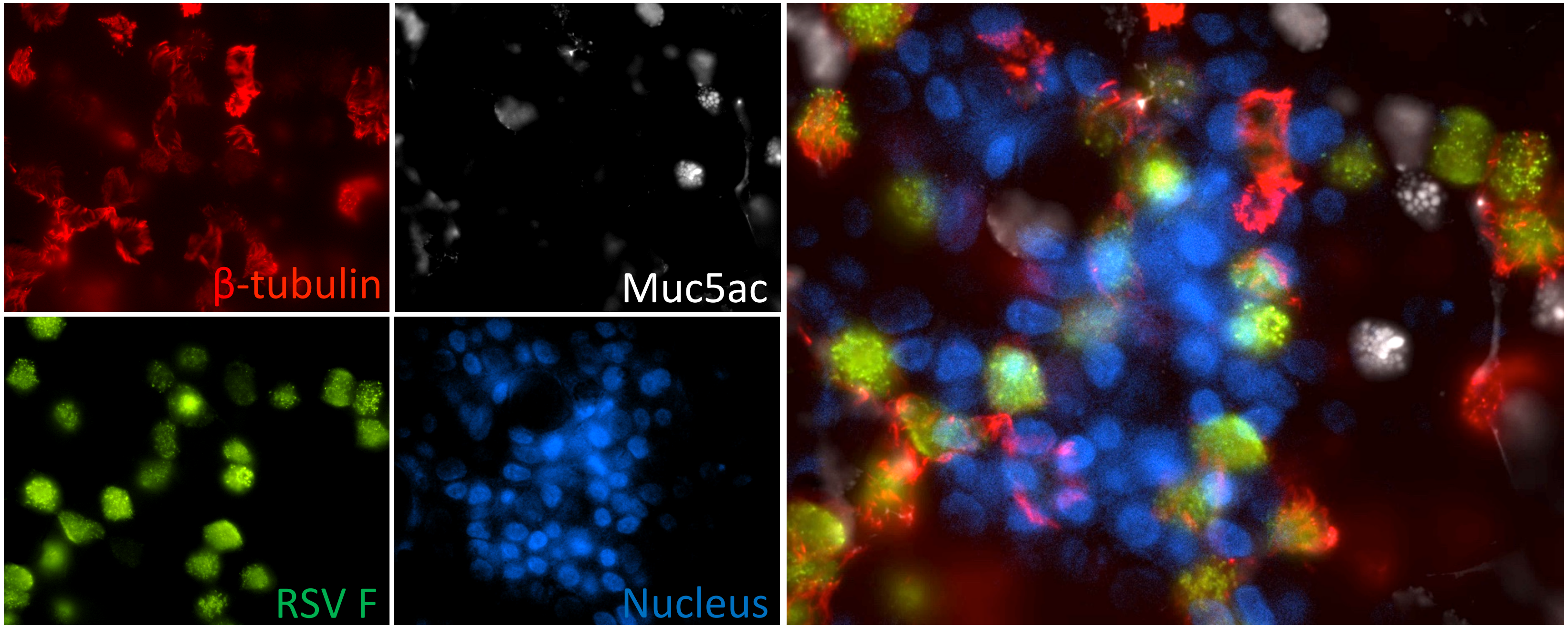

Figure S1: Individual channel for immunofluorescence staining of WD-PBECs infected with hRSV BT2a (figure 1B). The cultures were fixed at 120 hpi with PBS 4% paraformaldehyde (v/v; Sigma-Aldrich) and permeabilised with PBS 0.2% Triton X-100 (v/v; Thermo Fisher Scientific UK). After blocking with 0.4% BSA (in PBS, pH7.4) (Sigma Aldrich), cultures were stained with an anti-β-tubulin Cy3-conjugated monoclonal antibody (a ciliated cell marker) (Abcam, ab11309), an anti-RSV F 488-conjugated antibody (Millipore, MAB8262X), and a combination of rabbit anti-Muc5ac (a goblet cell marker) (Abcam, ab78660) and goat anti-rabbit IgG Alexa fluor 647-conjugated antibodies (Thermofisher, A21245). All antibodies were diluted in 0.4% BSA. Following treatment with DAPI-mounting medium (Vectashield, Vector Labs), Fluorescence was detected in the cultures by UV microscopy (Nikon Eclipse 90i) at a magnification x60 and each fluorophore channel is shown.

Figure S2:

A.

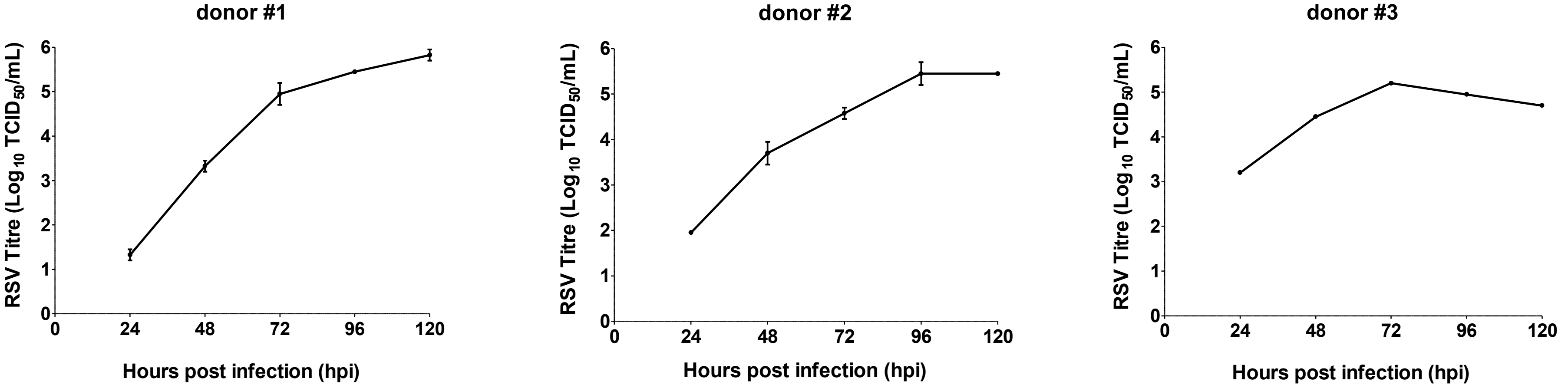

B.

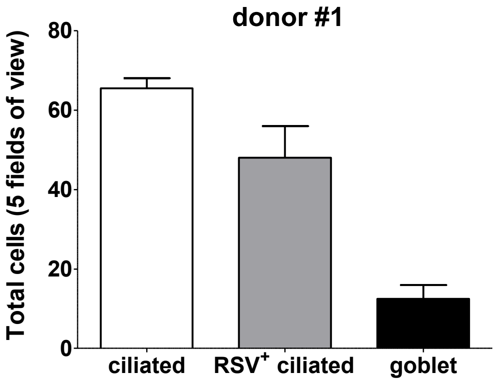

Figure S2: Virus growth kinetics in individual donor and distribution of cells. **(A)** Duplicate Transwells of well-differentiated primary paediatric cell cultures (WD-PBECs) from 3 donors were infected with RSV BT2a (MOI=0.1) or mock-infected. Apical rinses were harvested every 24 h and hRSV titres were titrated by $TCID_{50}$. **(B)** At 120 h post-infection (hpi) cultures were fixed with 4% paraformaldehyde. Ciliated and infected cells were visualized by immunofluorescence. The total number of ciliated cells and hRSV-infected ciliated cells were enumerated in 5 fields of view at x60 magnification (n=1 donor in duplicate).

Figure S3:

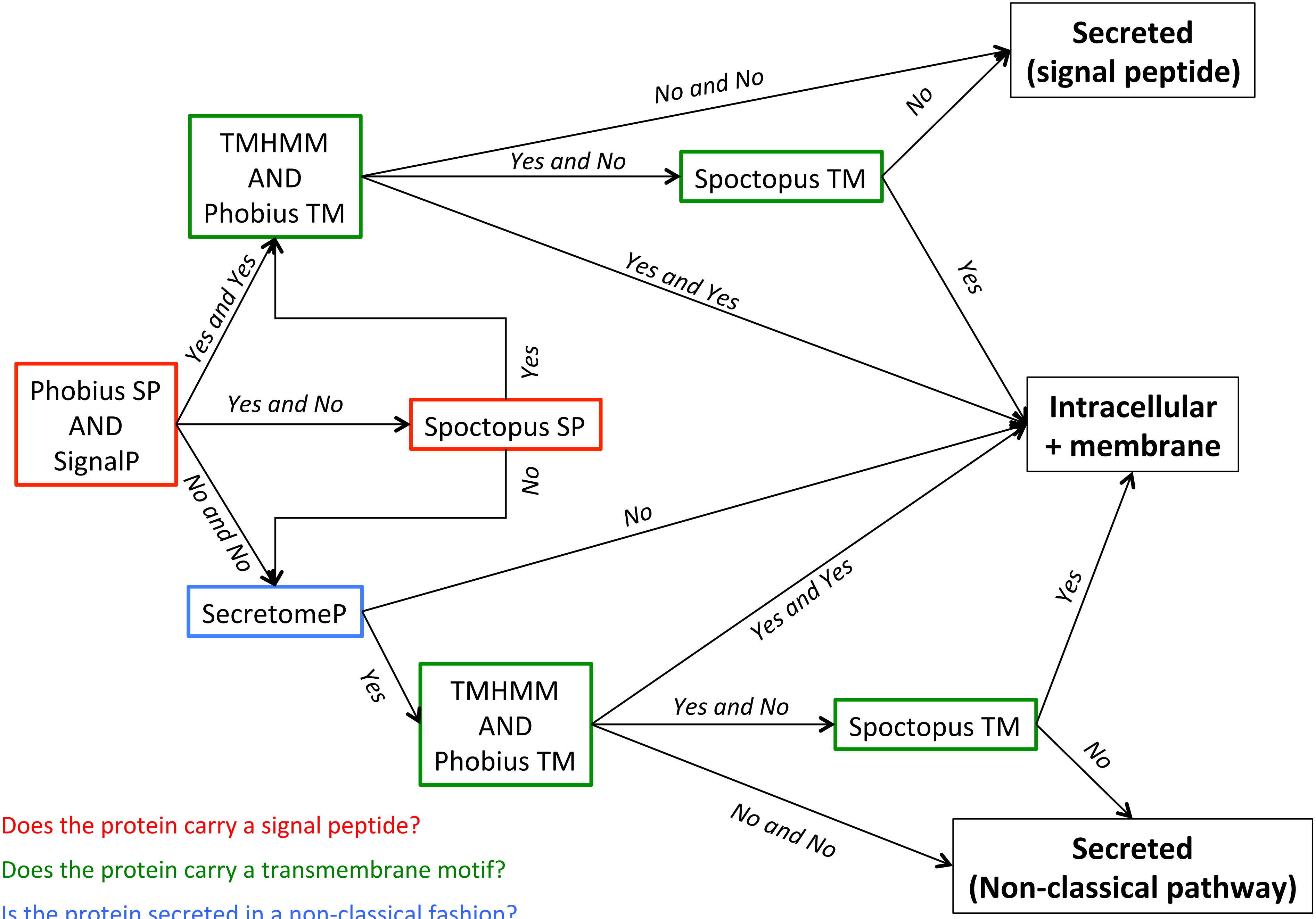

Does the protein carry a signal peptide?

Does the protein carry a transmembrane motif?

Is the protein secreted in a non-classical fashion?

Figure S3: Decision tree leading to the classification of host proteins between secreted via a signal peptide, secreted in a non-classical pathway, intracellular or membrane. Proteins positively identified in airway secretions of mock- and hRSV BT2a-infected cells were organized following a majority-decision based method (see Materials and Methods). Briefly, based on the amino acid sequence of a protein, six algorithmic methods were used to predict the presence of a signal peptide (SP) and/or a transmembrane region (TM). Phobius SP, SignalP and Spoctopus SP addressed the question of the presence of a SP (in red) whereas Phobius TM, Spoctopus TM and TMHMM focused on TMs (in green). If two out of three software positively identified (“Yes”) the presence of a SP independently of a TM in a protein, or *vice versa*, this protein fell under the signal peptide or membrane category, respectively. If a protein was predicted to carry both motifs simultaneously or no motif at all (“No”), by at least two algorithms, it was classified as membrane or run through SecretomeP (in blue), respectively. Positive prediction by SecretomeP (“Yes”) ranked the protein as likely to be secreted in a non-classical pathway or, inversely, as intracellular.

Figure S4:

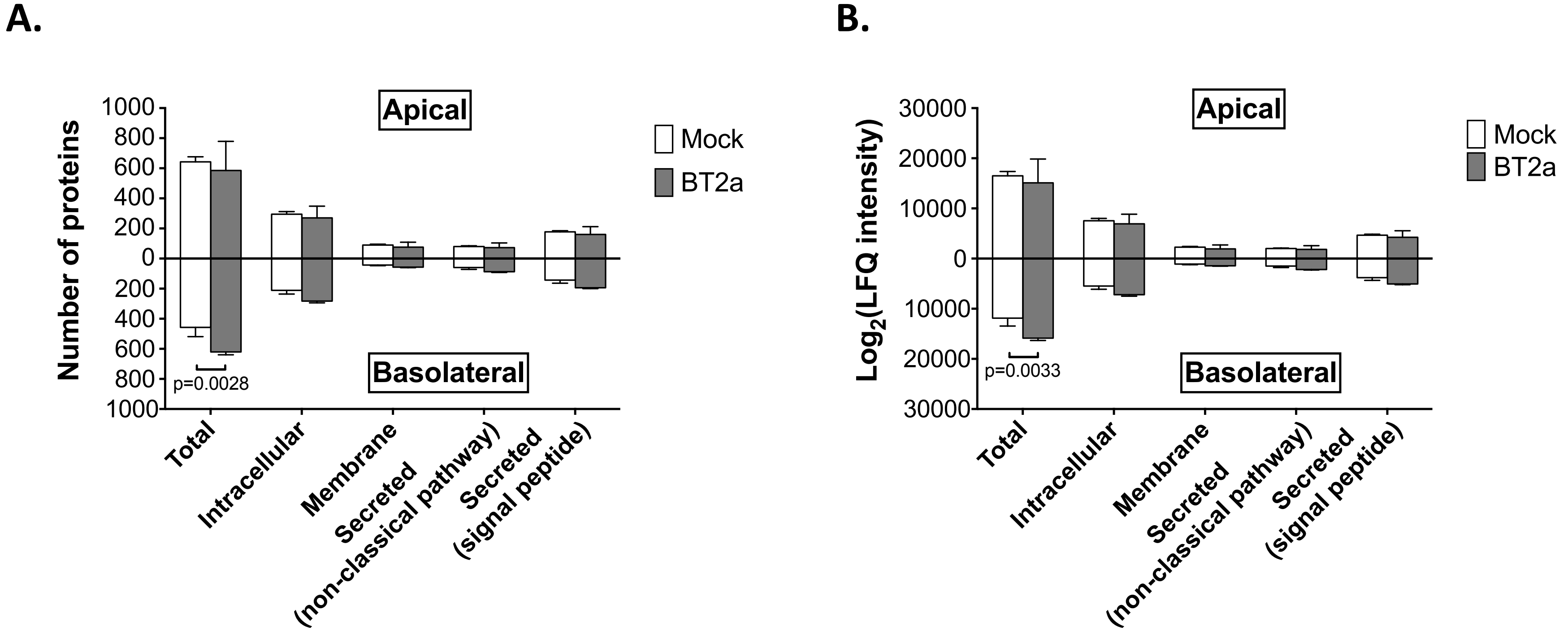

Figure S4: Apical and basolateral distribution of each protein category based on the protein number and abundance. Using specific software, proteins from control (white) and hRSV BT2a-infected (grey) cultures of each donor were stratified based on their predicted topologies (x-axis). For each category, the total number of proteins **(A)** and the total protein abundance **(B)** were graphically represented (y-axis) according to their apical (top) and basolateral (bottom) distributions. The cumulative number and abundance of each protein associated with a given protein category was determined.

Figure S5:

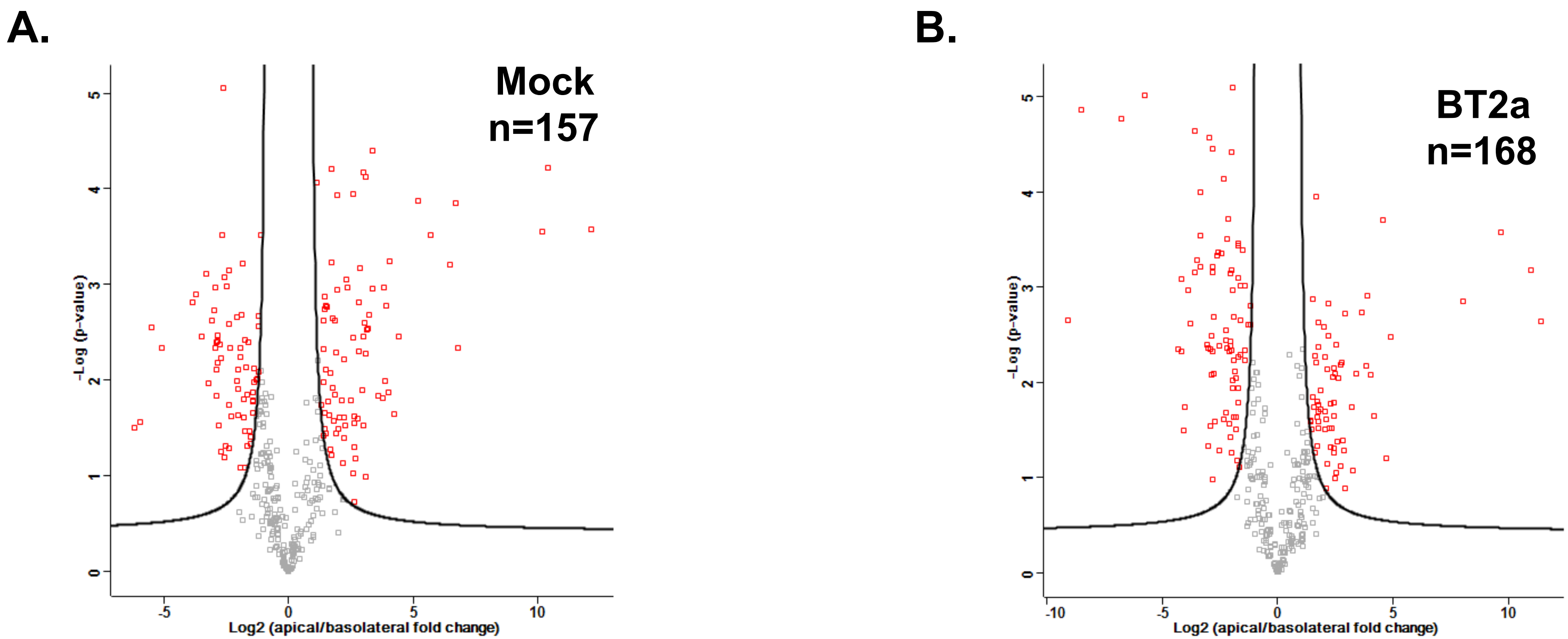

Figure S5: Proteins differentially abundant in the apical and basolateral compartments of mock and hRSV BT2a-infected WD-PBEC cultures. (A, B) Volcano plot representations of the proteins which were detected in both sides of mock **(A)** and hRSV BT2a-infected **(B)** cultures, respectively. Only proteins detected in both side in at least 2 replicates were taken into account. Each dot represents a protein plotted according to its apical/basolateral abundance ratio (x-axis) and an adjusted p-value (y-axis). Proteins with ratios ≥2 or ≤-2 and an adjusted p-value<0.05 were significantly more abundant in the apical or basolateral secretomes (red dots). Volcano plots were created using Perseus software. All proteins with a significant differential abundance under both conditions (n=229) were further plotted according to their apical/basolateral ratios in uninfected (x-axis) and infected (y-axis) WD-PBEC cultures (see figure 4C).

Figure S6:

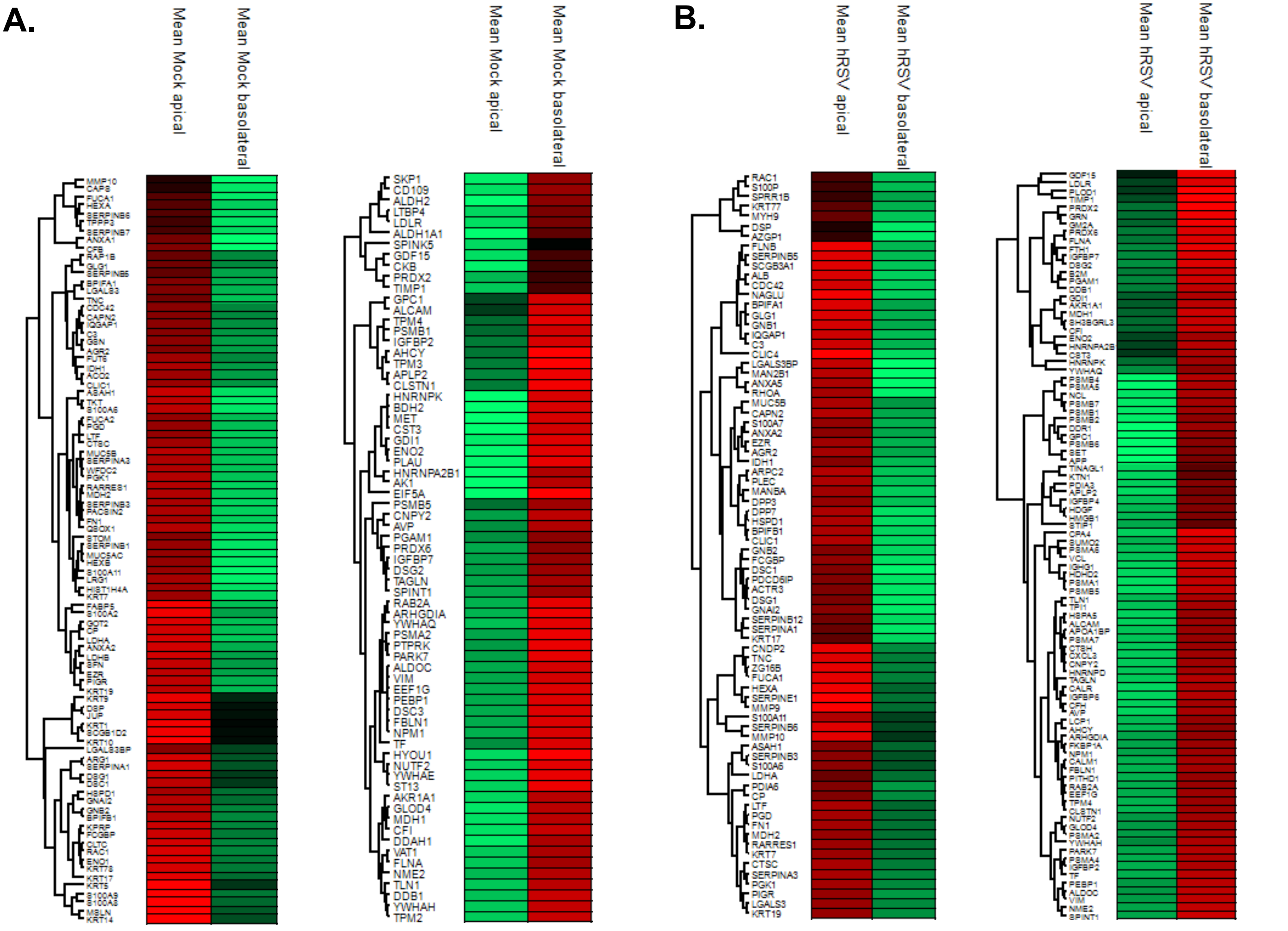
A.
Mean Mock apical
Mean Mock basolateral
Mean Mock apical
Mean Mock basolateral
B.
Mean hRSV apical
Mean hRSV basolateral
Mean hRSV apical
Mean hRSV basolateral

Figure S6: Hierarchical clustering of proteins whose abundance was significantly different between the apical and the basolateral side of (A) mock- and (B) hRSV-infected cultures. Only the 229 proteins identified by volcano plot analysis are shown (Figure E5, p-value < 0.05, fold change > 2). Log-2 transformed abundance values were averaged for each replicate then the mean was Z-score-normalized. The hierarchical clustering was performed with the rows indicating individual proteins and the columns individual replicate (green, low abundance; red, high abundance). The protein name is given for each row.

Figure S7:

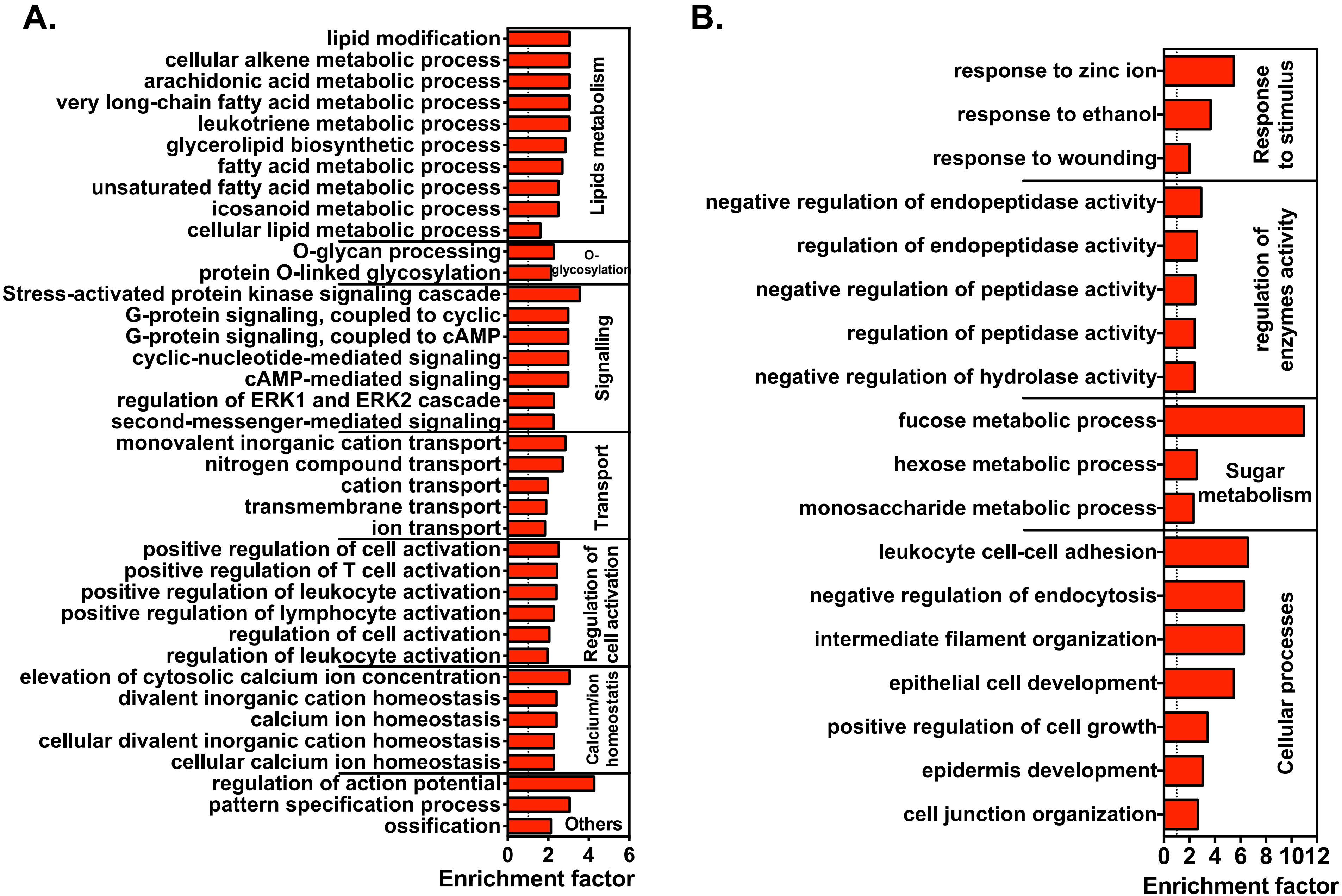
A.
lipid modification
cellular alkene metabolic process
arachidonic acid metabolic process
very long-chain fatty acid metabolic process
leukotriene metabolic process
glycerolipid biosynthetic process
fatty acid metabolic process
unsaturated fatty acid metabolic process
icosanoid metabolic process
cellular lipid metabolic process
Lipids metabolism
O-glycan processing
protein O-linked glycosylation
O-glycosylation
Stress-activated protein kinase signaling cascade
G-protein signaling, coupled to cyclic
G-protein signaling, coupled to cAMP
cyclic-nucleotide-mediated signaling
cAMP-mediated signaling
regulation of ERK1 and ERK2 cascade
second-messenger-mediated signaling
Signalling
monovalent inorganic cation transport
nitrogen compound transport
cation transport
transmembrane transport
ion transport
Transport
positive regulation of cell activation
positive regulation of T cell activation
positive regulation of leukocyte activation
positive regulation of lymphocyte activation
regulation of cell activation
regulation of leukocyte activation
Regulation of cell activation
elevation of cytosolic calcium ion concentration
divalent inorganic cation homeostasis
calcium ion homeostasis
cellular divalent inorganic cation homeostasis
cellular calcium ion homeostasis
Calcium/ion homeostatis
regulation of action potential
pattern specification process
ossification
Others
0 2 4 6
Enrichment factor
B.
response to zinc ion
response to ethanol
response to wounding
Response to stimulus
negative regulation of endopeptidase activity
regulation of endopeptidase activity
negative regulation of peptidase activity
regulation of peptidase activity
negative regulation of hydrolase activity
regulation of enzymes activity
fucose metabolic process
hexose metabolic process
monosaccharide metabolic process
Sugar metabolism
leukocyte cell-cell adhesion
negative regulation of endocytosis
intermediate filament organization
epithelial cell development
positive regulation of cell growth
epidermis development
cell junction organization
Cellular processes
0 2 4 6 8 10 12
Enrichment factor

Figure S7:

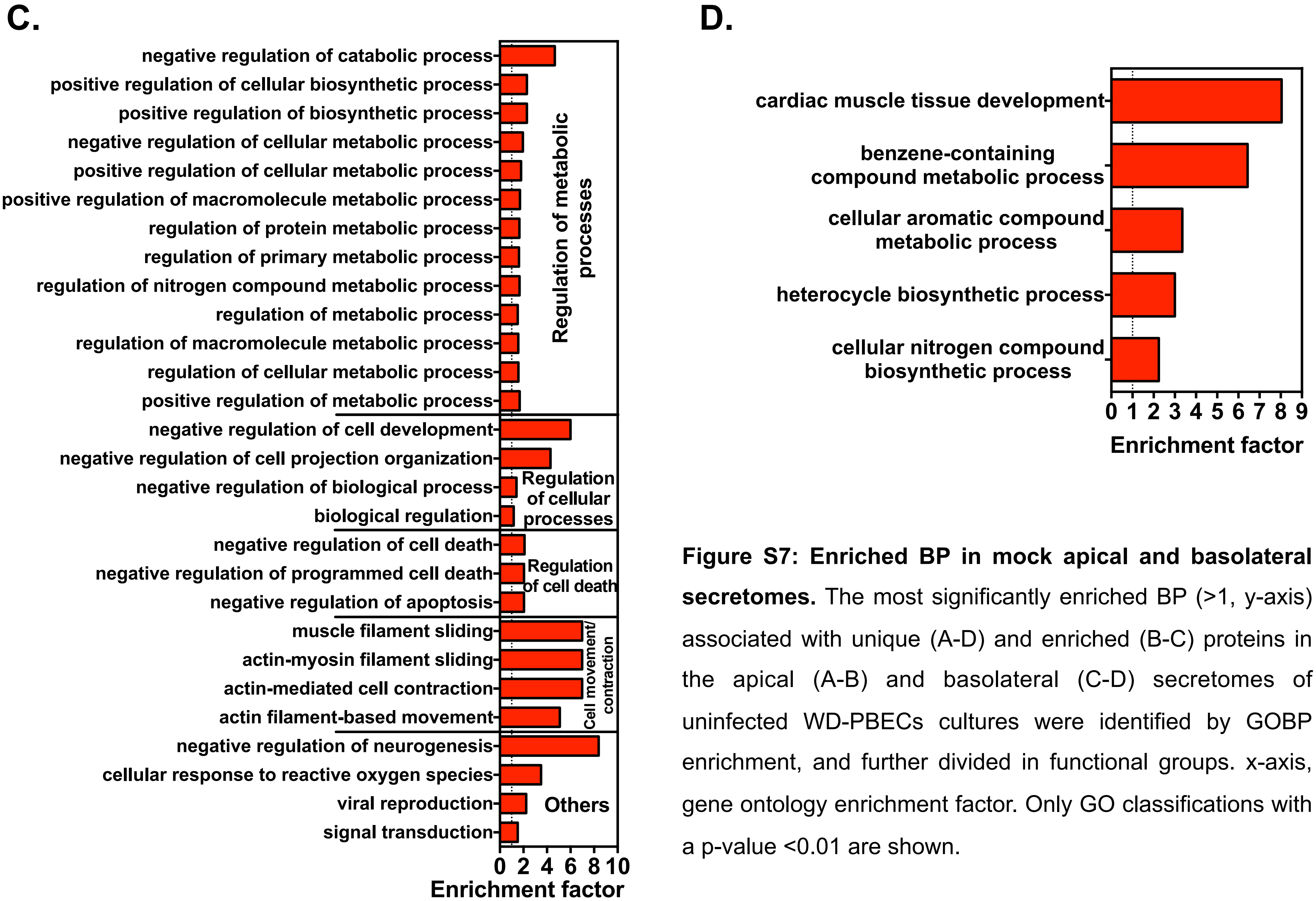

Figure S7: Enriched BP in mock apical and basolateral secretomes. The most significantly enriched BP (>1, y-axis) associated with unique (A-D) and enriched (B-C) proteins in the apical (A-B) and basolateral (C-D) secretomes of uninfected WD-PBECs cultures were identified by GOBP enrichment, and further divided in functional groups. x-axis, gene ontology enrichment factor. Only GO classifications with a p-value <0.01 are shown.

Figure S8:

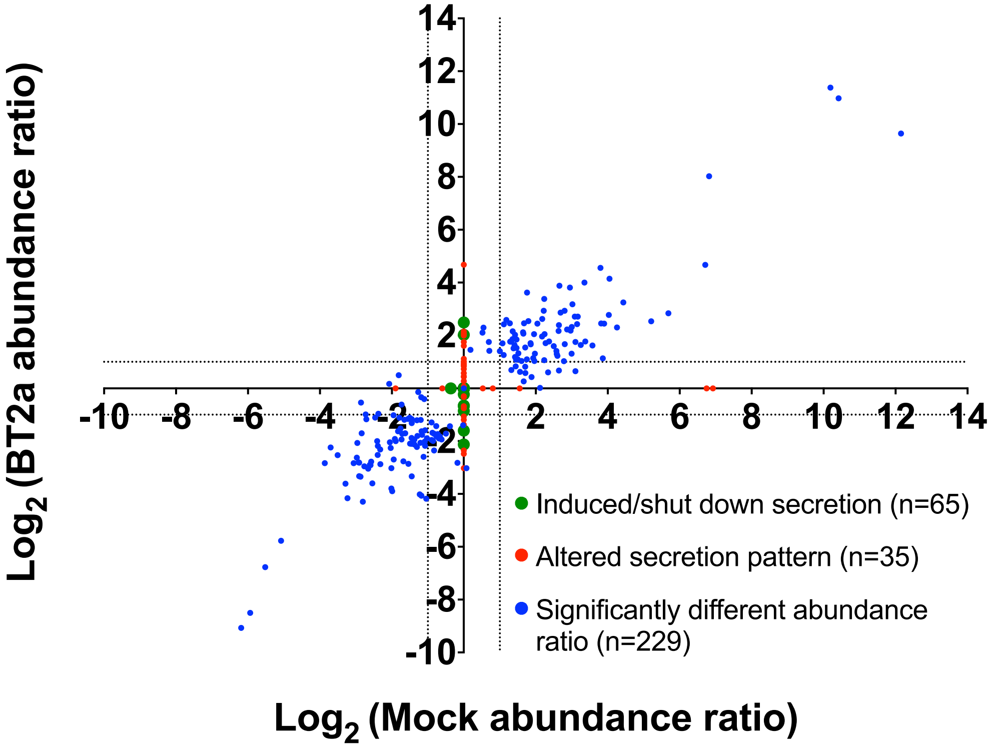

Figure S8: Apical/basolateral protein abundance ratios in the secretome of control and RSV-infected WD-PBECs cultures. Each dot represents a protein that was plotted based on its apical/basolateral abundance ratio in mock- (x-axis) and hRSV-infected (y-axis) WD-PBEC cultures. 229 proteins were found to be significantly more abundant in the apical or basolateral side, either under mock or infected conditions (blue dots), while 34 proteins were associated with a change in the polarity of secretion (red dots). The secretion of a further 65 proteins was either induced (n=53) or completely shut down (n=13) upon hRSV infection (green dots). Protein abundance was expressed as Log_2(Apical LFQ intensity/Basolateral LFQ intensity).

Figure S9:

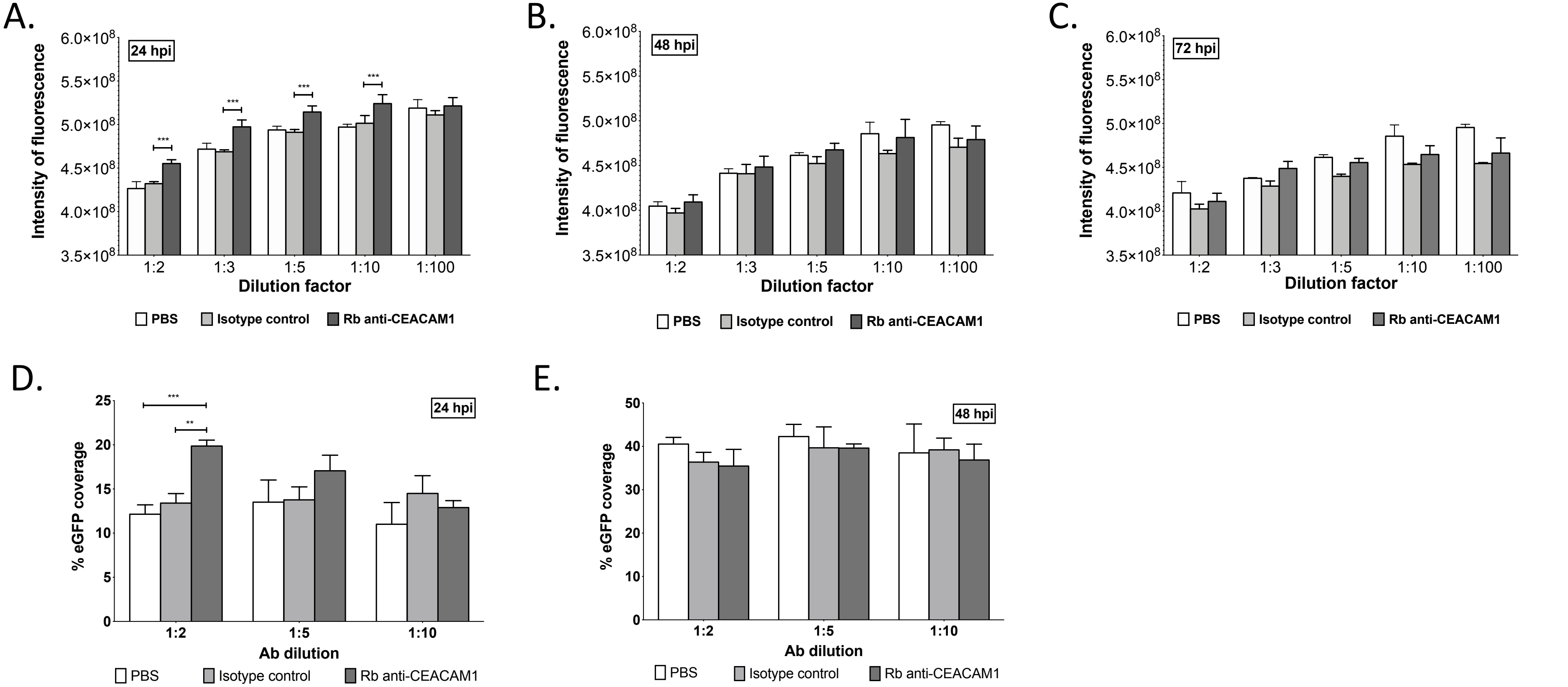

Figure S9: eGFP intensity and percentage coverage in BEAS-2B cells infected with hRSV/eGFP and incubated with a blocking antibody anti-CEACAM1. BEAS-2B cells were infected in triplicates with hRSV/eGFP (MOI=3) and incubated with serial dilutions of polyclonal rabbit antibodies anti-CEACAM1 and an isotype control (1:2, 1:3, 1:5, 1:10 and 1:100), against PBS-treated cells. Infection was followed by fluorescence imaging every 24 hpi (3 pictures taken per well) and the fluorescence was quantified with ImageJ software. The mean fluorescence intensity (A, B, C) and percentage of eGFP coverage (D, E) per field view was plotted for each dilution at 24, 48 and 72 hpi. Statistical analysis was assessed by a 2-way ANOVA (**=p-value<0.01; ***=p-value<0.001).
